# Jeffrey’s Insights: Next-generation sequencing for primary immunodeficiency diagnosis, 2019–2025

**DOI:** 10.70962/jhi.20250192

**Published:** 2026-10-20

**Authors:** Jessica Quinn, Enrique Fernández-Tabanera, Irene Perea-Romero, Miriam Leon-Otegui, María Moreno-Coca, Bibiana Palao, Luis Izquierdo, Vincenzo Cirigliano, Yi-Lee Ting, Larissa Albright, Vanessa Tenembaum, Ron Gladue, Jordan S. Orange, Fred Modell

**Affiliations:** 1 Jeffrey Modell Foundation, New York, NY, USA; 2 Veritas Intercontinental, Madrid, Spain; 3 Labcorp (formerly Invitae), San Francisco, CA, USA; 4 Children’s Hospital of Philadelphia, Department of Pediatrics, Perelman School of Medicine at the University of Pennsylvania, Philadelphia, PA, USA

## Abstract

Primary immunodeficiencies (PI), or inborn errors of immunity (IEI), are a clinically heterogeneous group of more than 550 monogenic disorders for which early molecular diagnosis is essential to guide management and improve outcomes. To address global barriers to genetic testing, the Jeffrey Modell Foundation implemented Jeffrey’s Insights, a no-cost next-generation sequencing (NGS) program delivered through the global Jeffrey Modell Centers Network. From 2019 to 2025, 5,991 patients across 59 countries underwent NGS testing, resulting in a molecular diagnostic yield of 22.7%. Molecular diagnostic yields were highest in Australia and New Zealand (40.9%), Asia (31.6%), and the Middle East and Africa (31.3%). Among 1,482 patients with clinician-reported follow-up, NGS altered diagnosis (38.1%), management (37.0%), treatment (32.8%), and genetic counseling (50.6%), and improved clinical outcomes (31.3%). Physicians reported on healthcare utilization and barriers to care prior to testing. These findings underscore the value of NGS as an available first-tier test for suspected PI and highlight the need for broader global access to genomic diagnostics.

## Teaser

In a global cohort of nearly 6,000 patients with suspected primary immunodeficiency, the Jeffrey Modell Foundation’s “Jeffrey’s Insights” next-generation sequencing (NGS) program demonstrates that, for trained clinical immunologists, NGS yields significant diagnostic and clinical value, improving patient care and outcomes.

## Introduction

Primary immunodeficiencies (PI), or inborn errors of immunity (IEI), represent an increasingly broad group of more than 550 monogenic disorders affecting immune development and/or function (1, 2, 3). These conditions can lead to recurrent and severe infections, autoimmunity, increased risk of malignancy, and may be life-threatening without appropriate diagnosis and timely intervention (3, 4, 5, 6, 7). PI may be more common than previously recognized, with population-level analyses reporting a prevalence of 1:1,200 and suggesting that up to 1% of individuals may have clinically relevant variants in immune-related genes or immune phenotypes (3, 8, 9, 10, 11).

Major advances in next-generation sequencing (NGS), including targeted, multigene panels, whole-exome sequencing (WES), and, more recently, whole-genome sequencing (WGS), have transformed the diagnosis of PI. These technologies have deepened the understanding of immune biology, expanded therapeutic strategies, and improved disease management and quality of life for many patients (7, 12, 13, 14, 15, 16, 17, 18). Nevertheless, prolonged and costly diagnostic delays, frequently referred to as the “diagnostic odyssey,” remain common. This process often involves repeated clinical evaluations, extensive and sometimes unnecessary testing, fragmented specialist care, undue stress, and avoidable morbidity, resulting in a significant burden not only to patients and families but also to healthcare systems worldwide (19, 20, 21, 22, 23).

Although NGS has demonstrated clear value in shortening the diagnostic odyssey and informing clinical care, significant barriers continue to hinder its widespread use (21, 24, 25, 26, 27, 28). These include restricted access to testing, insurance reimbursement challenges, high out-of-pocket costs, limited availability of immunology specialists, and persistent difficulties in variant interpretation and reporting (29). Consequently, many individuals with suspected PI remain undiagnosed or misdiagnosed, delaying effective treatment and contributing to preventable complications (22).

Accurate and timely molecular diagnosis is essential for guiding treatment, informing prognosis, supporting genetic counseling, and improving outcomes (22). Accordingly, NGS-based testing has increasingly been recognized as a cost-effective means of resolving the diagnostic odyssey for individuals with suspected PI (17, 18, 30, 31). Current targeted NGS panels include most known PI-associated genes and have shown strong diagnostic performance across multiple patient populations, with systematic reviews reporting molecular diagnostic yields of ∼15–45% (32, 33). Molecular results often have a direct clinical impact, including changes in diagnosis and patient management (13). Prior findings support that NGS-based diagnostic approaches yield a high percentage of results when guided by specialists experienced in recognizing the clinical and immunologic hallmarks of PI, thereby improving pretest probability and diagnostic efficiency (24). Together, these findings support the early integration of NGS into the diagnostic evaluation of patients with suspected PI.

The Jeffrey Modell Centers Network (JMCN) was established by the Jeffrey Modell Foundation (JMF) more than three decades ago to address the increasing need for specialized evaluation and coordinated care for patients with suspected or confirmed PI. The network was designed to provide infrastructure to promote collaboration among expert immunologists worldwide across clinical care, research, and education (20). Leveraging this established global network, JMF launched Jeffrey’s Insights in 2019 as a no-cost genetic sequencing program that enabled participating JMCN physicians to offer commercially available NGS testing to patients with suspected PI, providing an opportunity to evaluate the diagnostic yield and clinical impact of NGS when patients were selected for testing by expert clinical immunologists.

Given the expertise of participating clinical immunologists in selecting patients, we anticipated that molecular diagnostic yields would remain within the 15–45% range reported in the literature for targeted NGS (32) and would result in clinically meaningful changes in diagnosis and management. Accordingly, the primary aim of this initiative was to evaluate the diagnostic yield and clinical impact of NGS as a front-line diagnostic tool in cases where an expert immunologist had a high clinical index of suspicion for PI.

## Results

### Program participation and overall genetic findings

**Figure 1.**
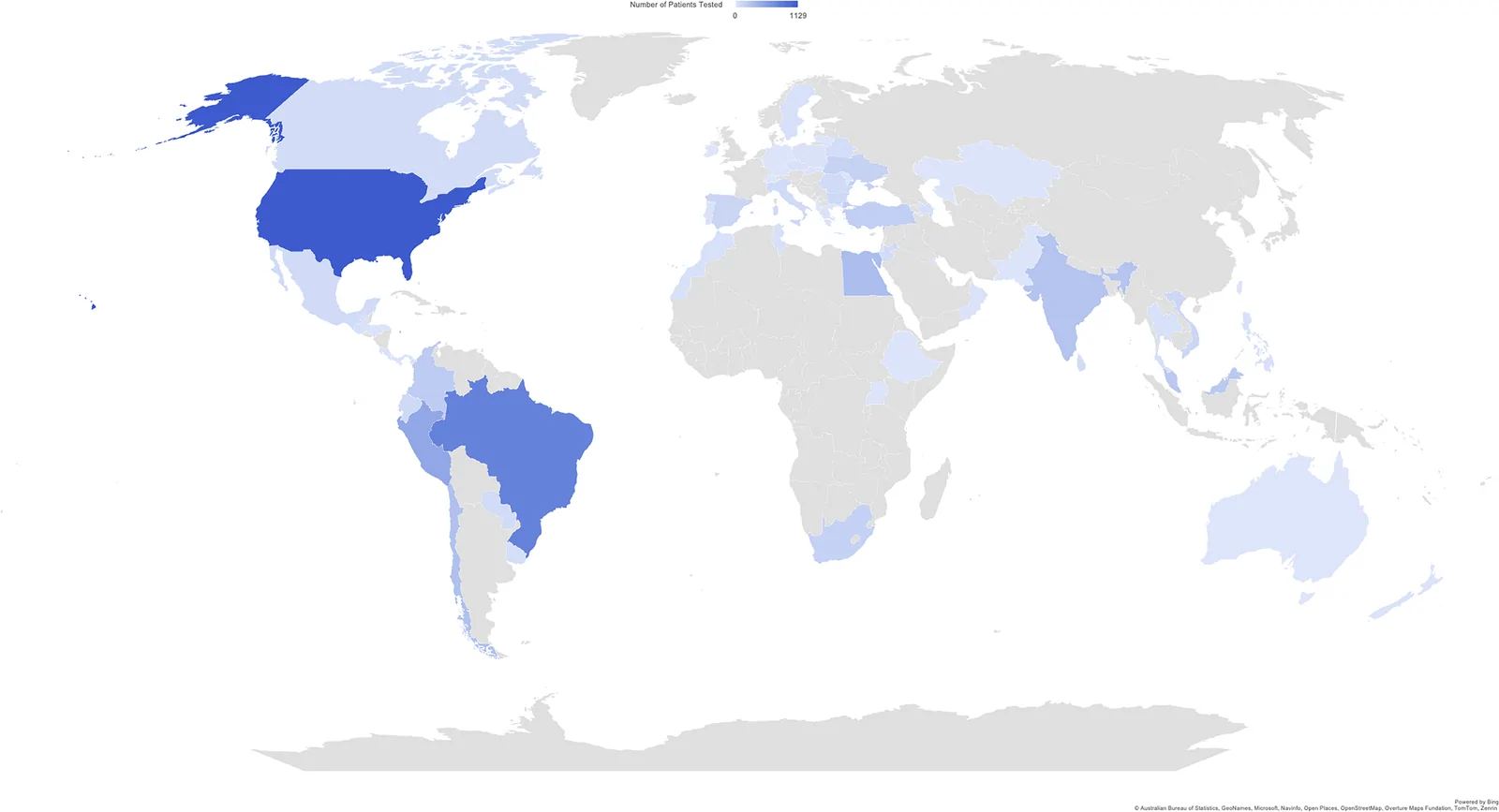
**Patients tested globally by country in the Jeffrey’s Insights program.** Countries are shaded from light to dark to correspond to the relative number of patients tested from low to high, respectively.

From January 2019 to June 2025, a total of 378 clinicians in the JMCN participated in this program, testing patients from 59 countries (Fig. 1 and Table S1).

**Figure 2.**
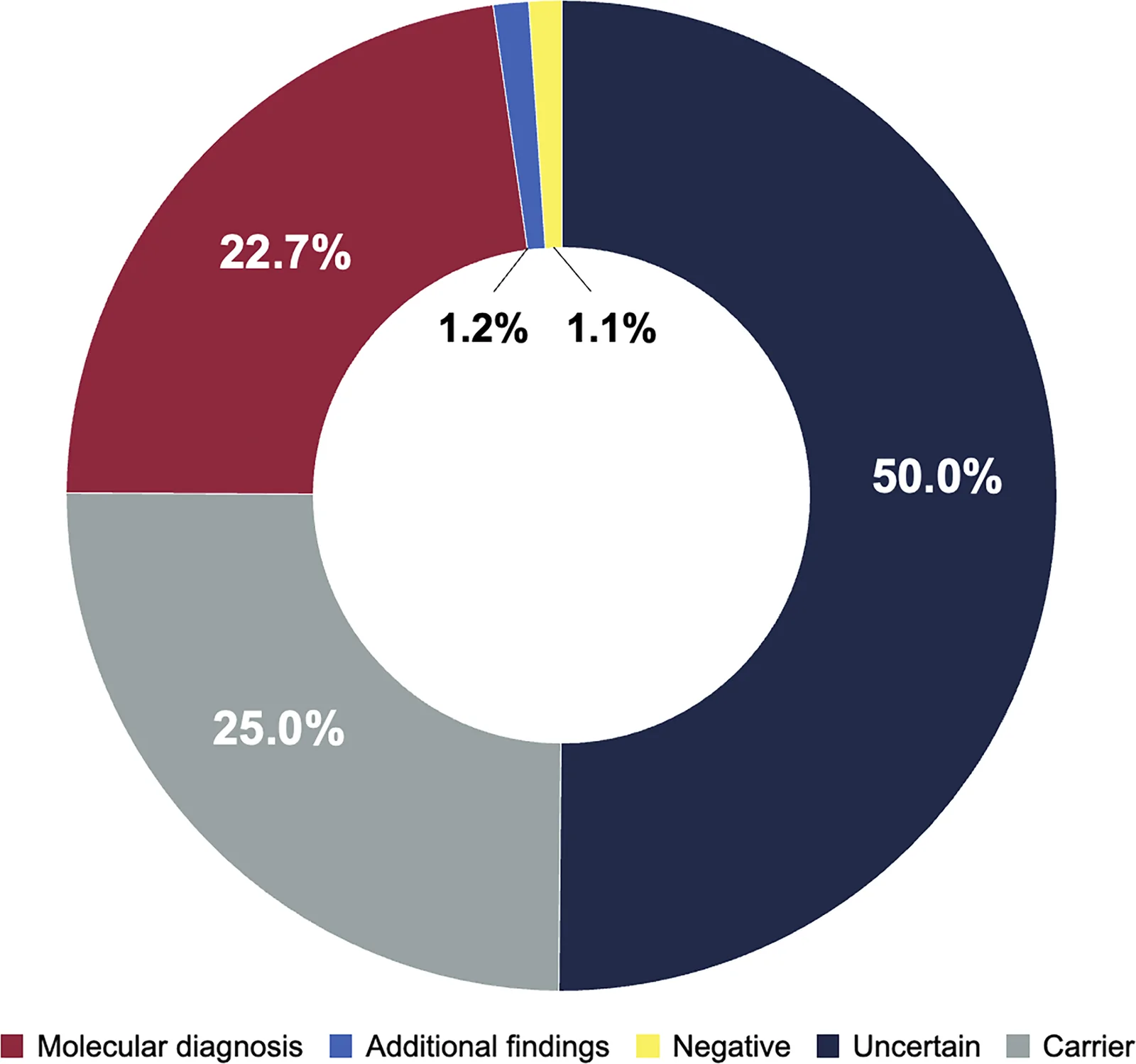
**The global molecular diagnostic yield of the Jeffrey’s Insights program.** Confirmed and likely molecular diagnoses were grouped together as molecular diagnosis. Variants in genes related to other clinically relevant non-PI conditions were included in the additional findings category. Carrier results are noted for individuals who had one variant in an autosomal recessive or X-linked recessive gene in a female. Uncertain results included findings of VUS only, and negative results reflected no P/LP or VUS variants detected.

During this period, a total of 5,991 patients were tested for suspected monogenic disorders, and 1,357 (22.7%) received a molecular diagnosis (Fig. 2). Additionally, 1,496 patients (25.0%) were carriers of autosomal recessive or X-linked conditions, and 69 patients (1.2%) had additional findings. The most common result was a report containing only variant(s) of uncertain significance (VUS) in 3,005 (50.2%) patients. Importantly, 74 patients had molecular diagnoses in more than one gene, with the majority of these having dual diagnoses. The most common combinations involved pathogenic variants in glucose-6-phosphate dehydrogenase deficiency, *DUOX2* (partial iodide organification defect), or TNF receptor superfamily member 13B, in combination with different PI-related genes. A complete list of molecular diagnoses attributed to known pathogenic variants, categorized by gene, according to the International Union of Immunological Societies (IUIS) Expert Committee classification of PI, is provided in Table S2.

A total of 57,346 nonunique reportable variants were identified, including variants identified in multiple patients, distributed across all IUIS categories (1), with the largest proportion represented by VUS (Table 1). At least one reportable genetic variant was found in 5,927 patients (98.9%). A total of 4,109 nonunique pathogenic (P)/likely pathogenic (LP)/risk (R) variants were identified. Of the 4,109 nonunique P/LP/R variants identified, 332 (8.1%) were P/LP copy number variants (CNVs). Of note, 199 (60.0%) of these 332 P/LP CNVs were sub-genic and could be missed by traditional microarrays.

**Table 1.** Number of nonunique variants identified by IUIS category

IUIS category (IUIS table)	P/LP positive (%)	P/LP carrier (%)	P/LP additional findings (%)	VUS (%)	Total
Immunodeficiencies affecting cellular and humoral immunity (Table 1)	271 (5.1)	161 (3.0)	1 (0.0)	4,929 (91.9)	5,362
Combined immunodeficiencies with associated or syndromic features (Table 2)	266 (3.1)	189 (2.2)	35 (0.4)	8,005 (94.2)	8,495
Predominantly antibody deficiencies (Table 3)	208 (7.1)	97 (3.3)	5 (0.2)	2,636 (89.5)	2,946
Diseases of immune dysregulation (Table 4)	202 (4.0)	106 (2.1)	1 (0.0)	4,714 (93.8)	5,023
Congenital defects of phagocyte number or function (Table 5)	197 (4.4)	323 (7.3)	2 (0.0)	3,917 (88.2)	4,439
Defects in intrinsic and innate immunity (Table 6)	108 (2.4)	65 (1.4)	0 (0.0)	4,398 (96.2)	4,571
Autoinflammatory disorders (Table 7)	144 (3.4)	219 (5.1)	0 (0.0)	3,932 (91.5)	4,295
Complement deficiencies (Table 8)	105 (4.1)	179 (6.9)	3 (0.1)	2,292 (88.9)	2,579
Bone marrow failure (Table 9)	44 (1.2)	88 (2.3)	20 (0.5)	3,638 (96.0)	3,790
Multiple IUIS tables	156 (10.2)	15 (1.0)	0 (0.0)	1,365 (88.9)	1,536
Not in IUIS, but has overlapping symptoms	175 (1.3)	699 (5.3)	25 (0.2)	12,411 (93.2)	13,310
Total	1,876 (3.3)	2,141 (3.7)	92 (0.2)	53,237 (92.8)	57,346

The number of nonunique P/LP, additional findings, carrier, and VUS variants that were identified in patients tested in the Jeffrey’s Insights program. Variants were attributed to the 2024 IUIS tables based on the gene that the variants were located in. P/LP variants were counted in the P/LP-positive column if the variant was sufficient for a diagnosis (seen in a gene with autosomal dominant inheritance or X-linked inheritance in a male) or if it was seen with a second P/LP/VUS variant in a gene with autosomal recessive inheritance. P/LP variants were counted in the P/LP carrier column when a single P/LP variant and no other variant was identified in an autosomal recessive gene. Percentages may not total 100% due to rounding.

### Molecular diagnostic yield by gene panel and geographic region

A total of 552 patients were tested using the 207 gene panel, 946 were tested using the 407 gene panel, 2,518 were tested using the 574 gene panel, 1,036 were tested using the 575 gene panel, and 939 were tested using the 576 gene panel (Table 2). The molecular diagnostic yield was not significantly different between patients tested on the 207 gene panel (20.5%), 407 gene panel (22.3%), 574 gene panel (22.6%), 575 gene panel (24.6%), and 576 gene panel (22.2%) (χ^2^(4) = 3.97, P = 0.409).

**Table 2.** Number of patients and molecular diagnostic yield by PI panel iteration

Panel	Dates of use	Number of patients (%)	Number of positive patients (diagnostic yield, %)
207 genes	January 2019–September 2020	552 (9.2)	113 (20.5)
407 genes	September 2020–March 2022	946 (15.8)	211 (22.3)
574 genes	March 2022–August 2023	2,518 (42.0)	570 (22.6)
575 genes	April 2023–April 2024	1,036 (17.3)	255 (24.6)
576 genes	April 2024–June 2025	939 (15.7)	208 (22.2)
Total	​	5,991	1,357 (22.7)

The total number of patients tested with each iteration of the PI panel is shown in the third column with the percentage of total cohort in parentheses. The subset of patients with confirmed or likely molecular diagnoses is shown in the fourth column with diagnostic yield in parentheses (P = 0.43). Percentages may not total 100% due to rounding.

**Figure 3.**
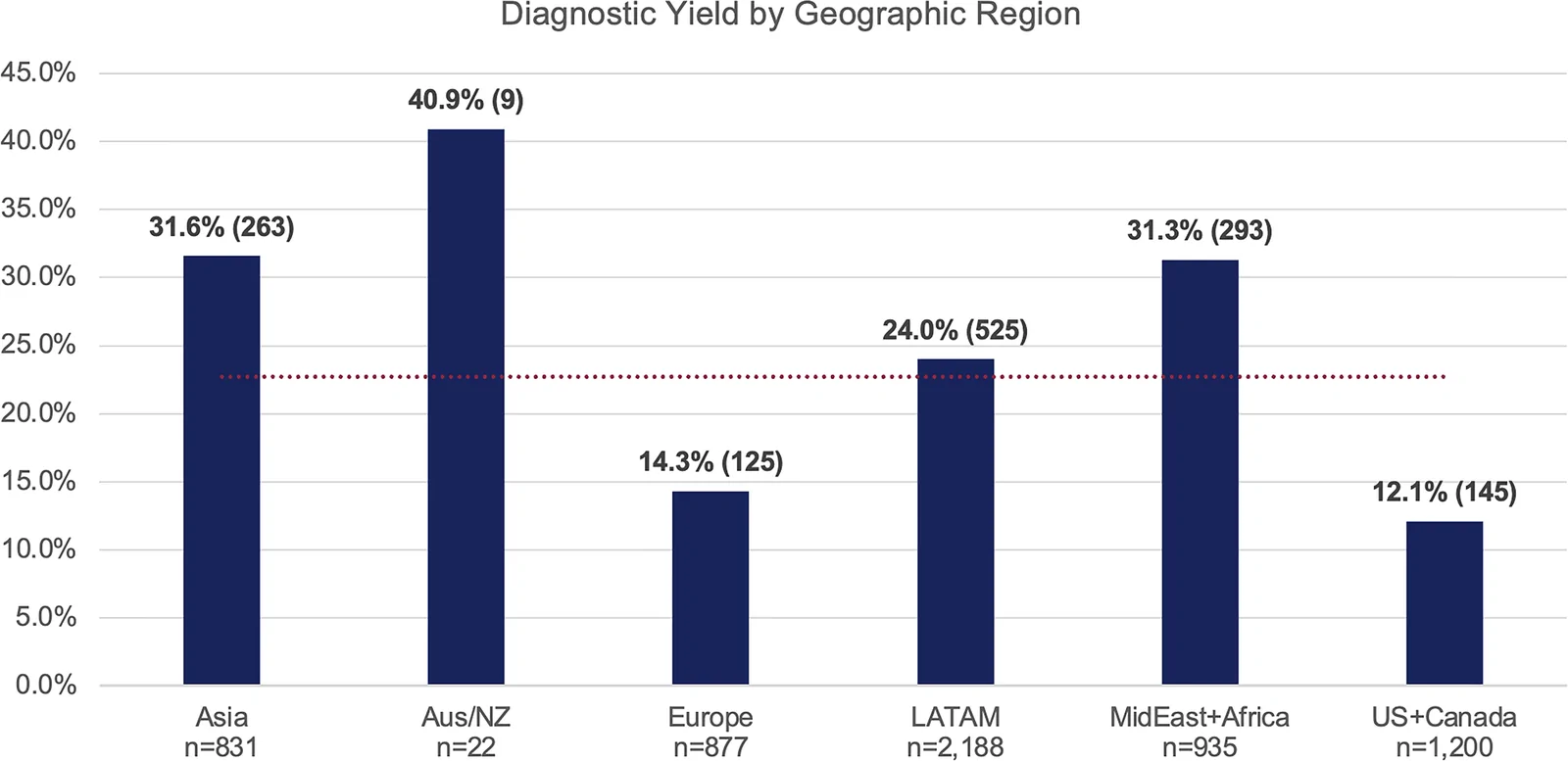
**Molecular diagnostic yield of the Jeffrey’s Insights program by geographic region** **, calculated the same way as the global molecular diagnostic yield.** The molecular diagnostic yield was calculated for patients from Asia, Australia and New Zealand (NZ), Europe, Latin America (LATAM), the Middle East (MidEast) and Africa, and the United States (US) and Canada. The dotted line represents the global molecular diagnostic yield (22.7%).

Molecular diagnostic yield varied by geographic region and was highest in Australia and New Zealand (40.9%), Asia (31.6%), the Middle East and Africa (31.3%), and Latin America (24.0%) compared with Europe (14.3%) and the US and Canada (12.1%) (χ^2^(5) = 197.9, P < 0.0001) (Fig. 3).

### Molecular diagnostic yield by patient demographics

Among the 5,991 patients evaluated, 43.8% were female, and 56.2% were male. The molecular diagnostic yield was higher in males (25.3%) compared with females (19.2%) (χ^2^(1) = 31.06, P < 0.0001). Patients were distributed across age groups as follows: 41.5% of patients were 5 years or younger, 31.0% were 6–17 years old, and 27.5% were adults aged 18 years and older. Molecular diagnostic yield was highest in the youngest age group (≤5 years), with 26.3% receiving a molecular diagnosis, followed by participants aged 6–17 years (20.7%) and adults (19.4%) (χ^2^(2) = 32.93, P < 0.0001). Of those receiving a molecular diagnosis, 76.5% were tested during the pediatric period (Table 3).

**Table 3.** Cohort demographics

​	Number of patients (%)	Number of positive patients (diagnostic yield, %)	P value
**Gender**	​	​	<0.0001
Female	2,625 (44)	505 (19)	​
Male	3,366 (56)	852 (25)	​
Total	5,991	1,357	​
**Age at testing (years)**	​	​	<0.0001
≤5	2,484 (42)	653 (26)	​
6–17	1,860 (31)	385 (21)	​
18+	1,647 (28)	319 (19)	​
TOTAL	5,991	1,357	​

Gender and age demographics of the total cohort are shown in the second column, both as the absolute number of patients as well as the percentage of the total cohort in parentheses. Gender and age demographics of the subset of patients with confirmed or likely molecular diagnoses are shown in the third column. The parentheses indicate the molecular diagnostic yield for each of the demographic categories. Percentages may not total 100% due to rounding.

### The JMF questionnaire

A total of 99 (26.2%) physicians from participating centers submitted complete questionnaires, representing 1,482 (24.7%) patients. Of these, the largest proportion of patients was from Latin America (42.1%), followed by Europe (23.3%), the US and Canada (18.6%), the Middle East and Africa (10.5%), Asia (5.3%), and Australia and New Zealand (0.3%).

**Figure 4.**
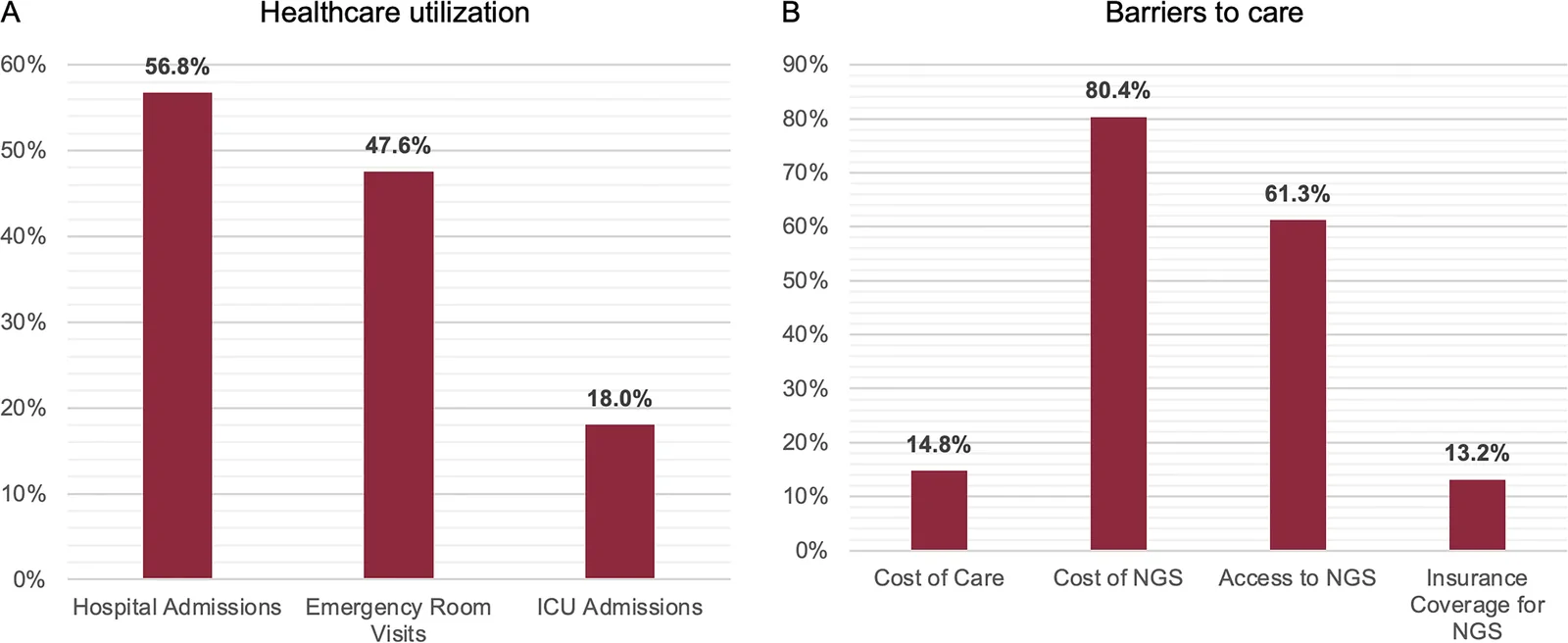
**Clinician responses to JMF questionnaire. (A and B)** Participating clinicians reported on patients (A) healthcare utilization and (B) barriers to care prior to NGS testing. ICU, intensive care unit.

Patients were reported to have visited a healthcare provider an average of 6.08 times over the preceding 12 mo. During this same period, 56.8% of patients experienced at least one hospitalization, 47.6% visited the emergency department, and 18.0% were admitted to the intensive care unit, with several patients having multiple admissions. 15% of patients reportedly did not seek medical care due to cost, and 80.4% did not pursue NGS testing for the same reason. Access to NGS was identified as a barrier for 61.3% of patients, and only 13.2% were reported to have insurance coverage for genetic testing (Fig. 4). Based on physician-reported estimates provided through the questionnaire, the average estimated annual cost of care before NGS testing was approximately $62,000 USD per patient. Reported cost varied substantially by geographic region, with estimated annual costs of $132,000 USD in Australia and New Zealand, $109,000 USD in the US and Canada, $60,000 USD in Latin America, $38,000 USD in the Middle East and Africa, $17,000 USD in Asia, and $13,000 USD in Europe. These estimates reflect physician-reported costs within diverse healthcare systems and should be interpreted in that context.

**Figure 5.**
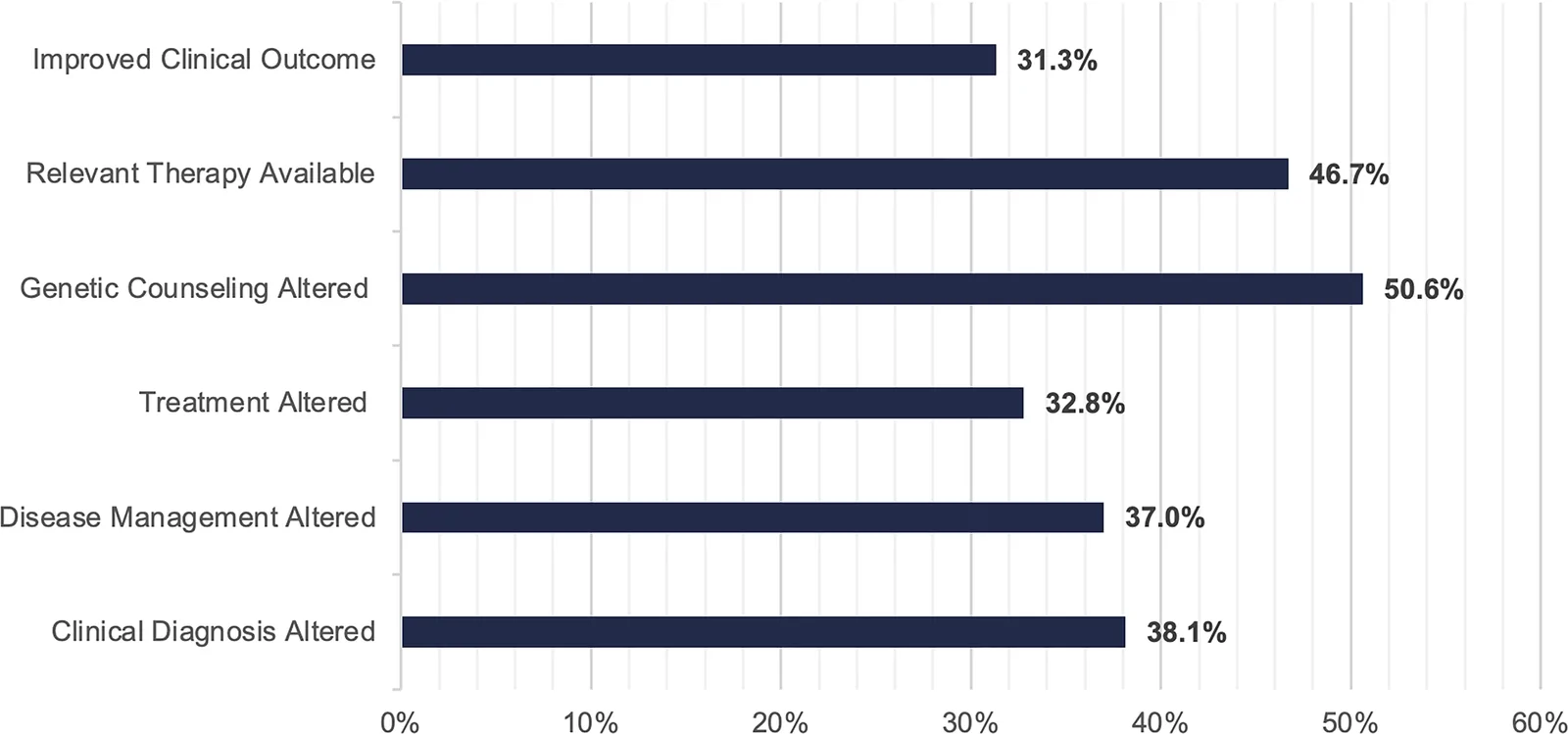
**Clinical impact of NGS testing.** Through a questionnaire, participating clinicians reported on the alteration rates of clinical diagnosis, disease management, treatment, and genetic counseling, as well as the percentage of patients with approved therapy available and improved clinical outcomes, after NGS testing.

Clinicians suspected a specific diagnosis in 74.0% of cases, but post-NGS results led to a change in the suspected diagnosis in 36.0% of these patients. Regardless of whether a definitive molecular diagnosis was achieved, NGS testing influenced clinical diagnosis in 38.1% of patients, disease management in 37.0%, treatment decisions in 32.8%, and genetic counseling in 50.6%. Additionally, testing identified at-risk or affected family members in 16.0% of cases. An approved therapy was identified for 46.7% of patients, and 31.3% were reported to have experienced improved clinical outcomes as a direct result of NGS findings (Fig. 5).

## Discussion

This global evaluation of JMF’s Jeffrey’s Insights program demonstrates the value of NGS as a frontline diagnostic tool for patients with suspected PI. Leveraging the broad reach and expertise of the JMCN, the program expanded access to molecular testing for nearly 6,000 patients across six continents, many of whom would otherwise face substantial barriers to genetic services. The overall molecular diagnostic yield of 22.7% reinforces that early NGS testing can substantially reduce, or even prevent, the prolonged diagnostic odyssey often experienced by patients with suspected PI. The program also demonstrates the successful real-world implementation of NGS across diverse healthcare systems and geographic regions.

A defining feature of the program was the reliance on expert clinical immunologists to identify patients with a high index of suspicion for PI. This clinician-driven approach yielded results consistent with previous studies (32, 33). Notably, the molecular diagnostic yield remained stable across five increasingly comprehensive gene panels, indicating that clinician-guided pretest probability was a stronger determinant of diagnostic success than panel size alone. These findings highlight the essential role of experienced specialists in recognizing subtle or evolving phenotypes of monogenic immune disorders and support broader access to NGS at their discretion.

The global scope of the program enabled evaluation of NGS performance across diverse populations. Molecular diagnostic yields varied by region, likely reflecting differences in population genetics, founder variants, consanguinity rates, referral patterns, and access to genetic testing and specialized immunology care (34, 35, 36, 37). Lower molecular diagnostic yields observed in North America and Europe may reflect broader prior availability of genetic testing, which likely resulted in earlier diagnosis of more straightforward cases outside this program and left a remaining cohort enriched for more complex conditions. Variations in patient populations, including a higher proportion of adults and disorders such as common variable immunodeficiency, may have also contributed. Improved characterization of regional differences may help optimize future panel design, variant interpretation, and deployment of genomic resources. Nevertheless, the consistently high rates of molecular diagnoses observed across all regions reaffirm that NGS is an essential diagnostic tool worldwide, particularly in settings where comprehensive immunologic evaluation or advanced genetic services are limited.

More than three-quarters of patients receiving a molecular diagnosis underwent testing during childhood, with pediatric patients demonstrating a higher molecular diagnostic yield than adults. Male patients also demonstrated a higher molecular diagnostic yield than female patients, likely due to the prevalence of X-linked immunodeficiencies. Together, these findings emphasize the importance of early genetic evaluation while highlighting differences in molecular diagnostic yield across patient subgroups.

The most common outcome of genetic testing in this cohort was the identification of only VUS (50.2%), highlighting a persistent challenge for patients, clinicians, and laboratories. Continued data sharing, functional studies, segregation analyses, and improved computational methods will be essential to improve variant interpretation (38). Most VUS are ultimately reclassified as benign, reinforcing current recommendations that they should not be used alone for clinical decision-making (39).

Despite the limited clinical actionability of isolated VUS findings, the JMF questionnaire demonstrated that NGS influenced patient care across multiple dimensions, regardless of whether a molecular diagnosis was obtained. Even nondiagnostic results informed clinical reasoning and refined management, helping avoid unnecessary interventions, guiding subsequent evaluation, and reducing patient and healthcare burden. The questionnaire also identified persistent barriers to NGS access, including limited availability and reimbursement, reinforcing the need to improve access to genetic testing worldwide. Frequent healthcare utilization highlighted the substantial clinical and economic burden of delayed diagnosis. Earlier NGS-guided diagnosis has the potential to reduce these encounters and associated costs (20, 24, 30, 40), supporting broader and earlier insurance coverage of diagnostic sequencing. These findings should, however, be interpreted in the context of the voluntary questionnaire, part of a programmatic quality improvement effort, completed by 26.2% of participating clinicians from a geographically uneven subset of the overall cohort. The generalizability of the findings may be limited because the molecular diagnostic yield varied by region. Nevertheless, the questionnaire provides valuable real-world insight into the clinical utility of NGS and the persistent barriers to implementation.

As NGS becomes increasingly integrated into routine clinical care, responsible management of genomic information is essential. Genomic data may have implications not only for the individual patient but also for biological relatives, underscoring the need for robust data governance, privacy protections, and responsible stewardship. Comprehensive sequencing raises ethical considerations related to incidental or secondary findings, long-term data storage, and the potential for periodic reanalysis as knowledge of disease-associated variants evolves. Continued attention to these issues will remain essential as global genomic sequencing programs continue to expand.

Collectively, these findings support NGS as a first-tier diagnostic test for patients with suspected PI, particularly when recommended by expert immunologists. Together with published evidence demonstrating potential healthcare savings exceeding $100,000 per patient annually (20, 24, 30, 31, 40), our findings support reducing barriers to testing, such as restrictive insurance policies, limited reimbursement, and outdated diagnostic algorithms. Although savings will vary across healthcare systems and regions, physicians reported substantial healthcare costs prior to NGS. These findings reinforce the clinical and economic value of earlier molecular diagnosis and support broader implementation of NGS, particularly in resource-constrained settings. Ultimately, expanding access to expert-guided genomic testing has the potential to shorten the diagnostic odyssey, enable precision medicine, and improve outcomes for patients with PI worldwide.

### Future directions

This global evaluation identifies several important directions for future investigation and program development. While WES and WGS have demonstrated higher diagnostic yields and offer advantages, including improved detection of challenging variant types and opportunities for reanalysis (41), the stable diagnostic yield observed across five increasingly comprehensive targeted panels indicates that panel-based testing remains a robust and effective first-line approach, especially in settings with limited resources. Future studies should focus on defining the optimal integration of targeted NGS, WES, and WGS within diagnostic workflows, including their roles in unresolved cases and longitudinal reanalysis. In addition, the program’s global reach highlights the need to better understand how population genetics, founder variants, ancestry, and regional referral patterns influence molecular diagnostic yield. Expanding regional databases and increasing representation of diverse populations will further improve variant interpretation and reduce uncertain findings.

Increasingly comprehensive genomic testing provides greater opportunities to identify multiple molecular diagnoses in a single patient. Although dual and triple molecular diagnoses were uncommon in this cohort, they reflect the evolution from single-gene testing to multigene panels, WES, and WGS, which have improved the detection of multiple concurrent disease-causing variants. Because functional validation was beyond the scope of this program, the contribution of individual variants in patients with multiple molecular findings remains uncertain. These findings raise important questions regarding blended phenotypes, oligogenic inheritance, and gene–gene interactions that warrant further investigation. In addition, ∼1% of patients had an “additional finding” in a gene unrelated to their primary PI presentation. While the clinical significance of these findings varied, and disease severity could not be determined, they emphasize the importance of careful interpretation, genetic counseling, and awareness of incidental or secondary findings as comprehensive genomic testing becomes increasingly common (42).

Future investigations incorporating standardized phenotype data will also be important to determine how diagnostic yield varies across clinical presentations and to identify phenotypes that remain underrecognized as suggestive of PI. These future directions highlight the continuing evolution of genomic diagnostics and the importance of integrating emerging technologies, expanding global genomic resources, and fostering international collaboration to further improve the diagnosis and care of patients with PI.

### Conclusion

The global Jeffrey’s Insights program demonstrates the substantial diagnostic and clinical value of NGS when applied early in the evaluation of patients with suspected PI. By leveraging the expertise of clinical immunologists across 59 countries, the program expanded access to genomic testing for nearly 6,000 patients and achieved an overall molecular diagnostic yield of 22.7%, with meaningful impacts on patient diagnosis, clinical management, treatment, and genetic counseling. These findings demonstrate that expert clinician-guided patient selection achieves high diagnostic efficiency across diverse populations and that NGS provides clinical value even when a definitive molecular diagnosis is not established.

Beyond its diagnostic performance, this program reflects the real-world implementation of NGS across diverse healthcare systems and highlights persistent global barriers to access. Collectively, these findings support the broader integration of NGS as a first-line diagnostic test for patients with suspected PI when recommended by qualified experts and reinforce the importance of early molecular diagnosis in improving patient outcomes worldwide. Continued collaboration among clinicians, laboratories, researchers, patient advocacy organizations, policymakers, and healthcare systems will be essential to advance access to genomic medicine and precision care for patients with PI.

## Materials and methods

### JMCN

The global JMCN was established by JMF >3 decades ago to improve access to specialized evaluation and coordinated care for individuals with suspected or confirmed PI. The network serves as a global platform for collaboration among expert immunologists, advancing clinical care, research, and education (20). The JMCN now includes more than 930 physicians across 500 institutions in 345 cities and 93 countries, spanning six continents. Collectively, network clinicians care for more than 265,000 patients, over half of whom lack a confirmed molecular diagnosis. The JMCN served as the clinical infrastructure for Jeffrey’s Insights by providing direct access to expert clinical immunologists who manage large cohorts of patients presenting with characteristic phenotypes of PI but without a genetic diagnosis.

### Global Jeffrey’s Insights program

In January 2019, JMF initiated Jeffrey’s Insights, a pilot no-cost genetic sequencing program, by inviting clinical immunologists within the JMCN across 10 participating countries to offer commercially available NGS to patients with suspected PI, based on the clinical immunologist’s assessment of a high likelihood of obtaining a result (43). The primary objective of the pilot was to evaluate whether NGS could improve diagnostic precision, inform clinical management, and reduce the diagnostic odyssey that patients with PI frequently experience. Following encouraging pilot results, Jeffrey’s Insights expanded globally in early 2020 to include all JMCN centers, substantially increasing access to NGS for patients with suspected PI (24).

### Description of testing services

JMF partnered with two accredited clinical diagnostic laboratories. Samples were initially sent by participating physicians to Invitae (now part of Labcorp) from January 2019 to August 2023, then to Veritas Intercontinental from April 2023 to June 2025. The PI panel, provided in partnership with Invitae, initially included 207 genes and was expanded to 407 in September 2020 and to 574 in March 2022. Results were previously published as part of a pilot (43) and expanded global cohort (24). Veritas developed an initial PI panel for Jeffrey’s Insights that included 575 genes, which was then expanded to 576 genes in April 2024. A list of the genes included on each panel is provided in Table S3. To compare the molecular diagnostic yield between panels, we used a chi-squared test with alpha P < 0.05. All other aspects of the program protocol, including patient eligibility and informed consent, remained as previously described (24, 43). All genomic and associated clinical information were managed in accordance with applicable privacy regulations and informed consent procedures. Patient information was de-identified prior to analysis and sharing, and only authorized personnel at Veritas and Invitae (now a part of Labcorp) had access to identifiable information where required for clinical interpretation.

### Patient selection

Patients were selected for NGS by clinical immunologists within the JMCN based on clinical judgment, specifically the assessment of a high likelihood of obtaining a diagnostically meaningful result, with no formal inclusion or exclusion criteria. The decision to pursue testing was informed by the treating immunologist’s evaluation and, in many cases, was supported by clinical features suggestive of PI. These features included (1) a confirmed clinical diagnosis of PI without prior molecular testing; (2) abnormal or inconclusive newborn screening results suggestive of PI without an established genetic etiology; and/or (3) clinical findings consistent with PI, including those aligned with the JMF 10 Warning Signs of PI (https://www.info4pi.org/information-booth/10-warning-signs/).

### Sample collection

Physicians submitted blood, saliva, and extracted genomic DNA samples from patients, which were shipped to the testing laboratories via a courier service for the detection of single nucleotide variants, indels, and exon-level deletions and duplications using NGS technology, as previously described (44, 45, 46, 47).

### Variant interpretation and classification

Variant interpretation was performed by the clinical laboratories providing testing, using multidisciplinary teams of expert PhD scientists, physicians, and genetic counselors who reviewed sequence data and interpreted variants in accordance with established clinical laboratory standards.

Variants were classified using the guidelines from the American College of Medical Genetics and Genomics (ACMG) and the Association for Molecular Pathology (AMP) (39) at Veritas, or a validated framework based on the ACMG/AMP guidelines at Invitae (now part of Labcorp) (48). Variants were classified as benign (B), likely B, VUS, LP, or P. Additionally, R variants were interpreted by Veritas according to ClinGen’s and ACMG recommendations (49, 50).

A detailed description of the variant classifications and inheritance patterns considered clinically relevant can be found in Table 4. A “confirmed” or “likely molecular diagnosis,” herein “molecular diagnosis,” referred to P/LP variant(s) and certain high-suspicion VUS that matched the inheritance pattern of the gene and PI condition. A heterozygous P/LP variant in a gene with autosomal recessive inheritance or X-linked recessive inheritance in a female patient was considered a “carrier” of the specific condition. The presence of P/LP/R variant(s) in genes related to other non-PI relevant conditions was considered as “additional findings” (e.g., a heterozygous P/LP variant identified in the *ATM* gene, which is associated with an autosomal dominant cancer predisposition and was unlikely related to the indication for referral).

**Table 4.** Clinical relevance of genetic test results

Confirmed or likely molecular diagnosis	Carrier status	Uncertain	Additional findings
1 heterozygous P/LP allele in AD gene	1 heterozygous P/LP/R allele in AR gene	Only VUS reported	1 heterozygous P/LP/R allele in AD gene related to non-PI conditions
1 hemizygous P/LP allele in XL gene in male	1 heterozygous P/LP allele in XL gene in female	​	​
2 heterozygous or 1 homozygous P/LP alleles in AR genes	​	​	​
1 homozygous VUS allele in AR genes with a high clinical relevance^a^	​	​	​
1 heterozygous P/LP allele and 1 VUS in AR genes	​	​	​

Columns summarize the potential genotypes identified among patients related to their clinical presentation. For heterozygous P/LP variants in genes linked to both AD and AR inheritance, variant-specific information was used to determine whether the finding represented an AD diagnosis or AR carrier status. AD, autosomal dominant; AR, autosomal recessive; LP, likely pathogenic; P, pathogenic; R, risk variant; XL, X-linked; VUS, variant(s) of uncertain significance.

a Applies to Veritas only.

For testing performed by Invitae (now a part of Labcorp), variant classification and the evidence used were deposited in ClinVar. For testing performed by Veritas, in addition to the ACMG/AMP guidelines, an independent interpretation framework for risk alleles was used where appropriate, and an initial filter using a minor allele frequency threshold of 1% was applied. However, variants previously classified as pathogenic or LP in ClinVar were reviewed regardless of allele frequency. Final clinical reports were provided to the ordering immunologist for interpretation within the context of the patient’s phenotype.

### The JMF questionnaire

**Figure S1.**
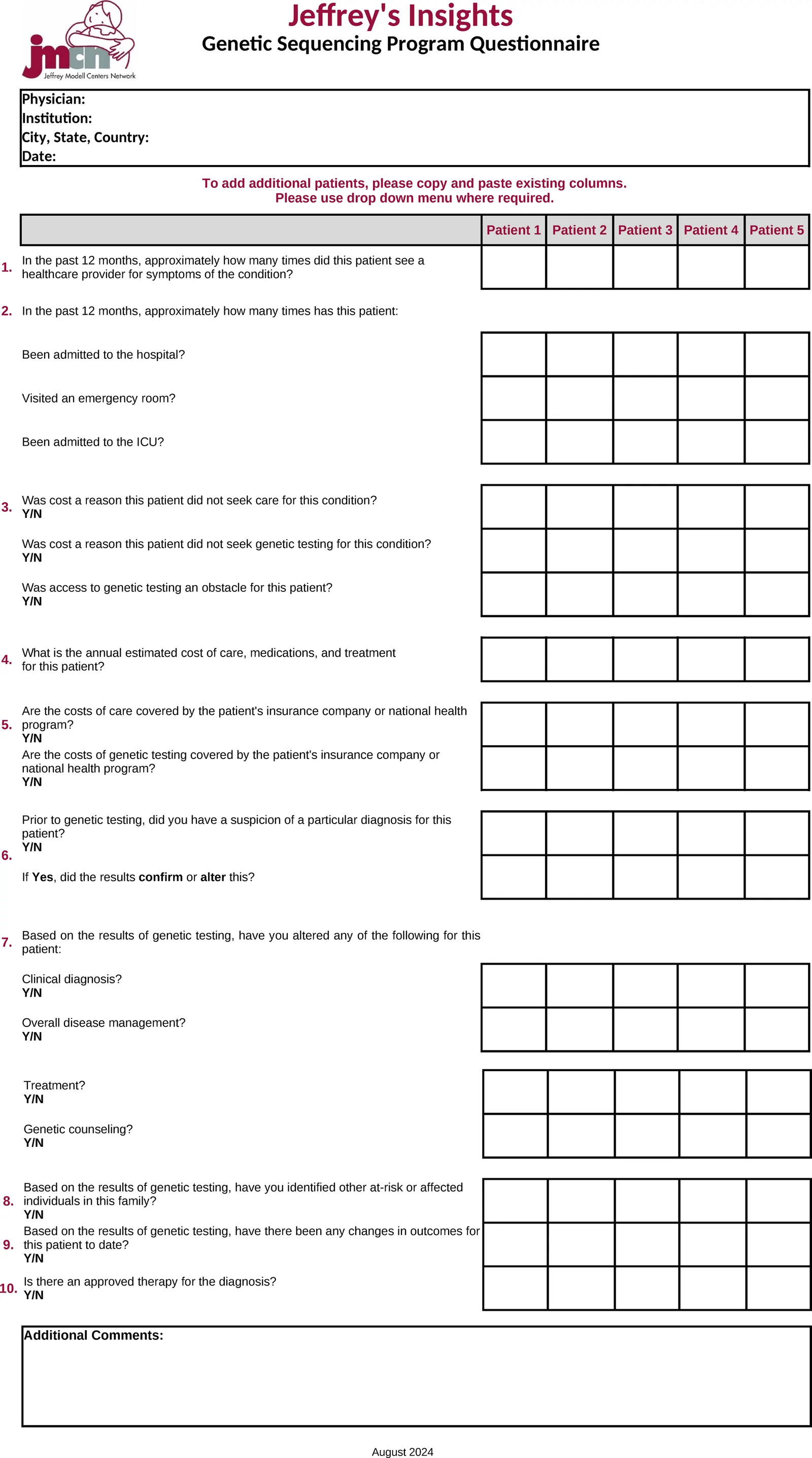
JMF physician questionnaire.

We provided an optional questionnaire to participating clinicians, requesting information on challenges in care and access to NGS, any subsequent changes in clinical management following testing, and estimates of the annual cost of care prior to NGS (Fig. S1). The “Additional Comments” field included in the questionnaire was not analyzed or incorporated into this manuscript. Originally intended for program improvement, this information proved valuable and was used to characterize the clinical relevance and practical impact of NGS in patients with suspected PI.

### Online supplemental material

Supplementary information includes the JMF physician questionnaire (Fig. S1), a table summarizing the number of patients tested by country (Table S1), a table listing the variants associated with positive results (Table S2), and a table listing the genes included on each iteration of the PI panel used in the Jeffrey’s Insights program (Table S3).

## Supplementary Material

Table S1 shows the number of patients tested by country.

Table S2 shows a list of variants associated with a positive result.

Table S3 shows the genes included on each iteration of the PI panel used in the Jeffrey’s Insights program.

## Data Availability

Data are available in the article itself and its supplementary material. Further inquiries can be directed to the corresponding author.
