# Supplementary material for: Jeffrey’s Insights: Next-generation sequencing for primary immunodeficiency diagnosis, 2019–2025
Source: J Hum Immun. 2026 Oct 20;3(1):e20250192. doi: 10.70962/jhi.20250192 (PMC13682337; doi:10.70962/jhi.20250192)
Supplement: Supplementary file 1 [file jhi_20250192_tables1.docx]

| **Supplementary Table 1. The number of patients tested by country** | |
| --- | --- |
| **Country** | **Number of Patients Tested** |
| Albania | <5 |
| Australia | <5 |
| Azerbaijan | 47 |
| Belarus | 64 |
| Brazil | 789 |
| Bulgaria | 105 |
| Canada | 71 |
| Chile | 293 |
| Colombia | 207 |
| Costa Rica | 24 |
| Czechia | 42 |
| Ecuador | 96 |
| Egypt | 320 |
| Ethiopia | 6 |
| Germany | 7 |
| Great Britain | 48 |
| Greece | 14 |
| Guatemala | 36 |
| Honduras | 11 |
| India | 268 |
| Ireland | <5 |
| Israel | 66 |
| Italy | 87 |
| Jordan | 86 |
| Kazakhstan | 5 |
| Kuwait | 12 |
| Lebanon | <5 |
| Lithuania | 13 |
| Malaysia | 227 |
| Mexico | 85 |
| Moldova | 25 |
| Morocco | 37 |
| New Zealand | 18 |
| Oman | 20 |
| Pakistan | 5 |
| Panama | 18 |
| Paraguay | 84 |
| Peru | 493 |
| Philippines | 32 |
| Poland | 22 |
| Portugal | 20 |
| Romania | 87 |
| Singapore | 26 |
| Slovakia | 15 |
| South Africa | 155 |
| Spain | 126 |
| Sri Lanka | 65 |
| Sweden | 37 |
| Switzerland | <5 |
| Taiwan | 7 |
| Thailand | 38 |
| Tunisia | 39 |
| Turkey | 181 |
| Uganda | 7 |
| Ukraine | 157 |
| United Arab Emirates | <5 |
| United States | 1,129 |
| Uruguay | 52 |
| Vietnam | 111 |
