# Supplementary material for: Jeffrey’s Insights: Next-generation sequencing for primary immunodeficiency diagnosis, 2019–2025
Source: J Hum Immun. 2026 Oct 20;3(1):e20250192. doi: 10.70962/jhi.20250192 (PMC13682337; doi:10.70962/jhi.20250192)
Supplement: Supplementary file 2 [file jhi_20250192_tables3.docx]

| **Supplementary Table 3. Genes included on each iteration of the PI panel used in the Jeffrey’s Insights program** | | | | |
| --- | --- | --- | --- | --- |
| **207 gene panel** | **407 gene panel** | **574 gene panel** | **575 gene panel** | **576 gene panel** |
| **Jan 2019-Sept 2020** | **Sept 2020-March 2022** | **March 2022-Aug 2023** | **April 2023-April 2024** | **April 2024-June 2025** |
| *ACD* | *ACD* | *ABCB7* | *ABCB7* | *ABCB7* |
| *ACP5* | *ACP5* | *ABCG5* | *ABCG5* | *ABCG5* |
| *ACTB* | *ACTB* | *ABCG8* | *ABCG8* | *ABCG8* |
| *ADA* | *ADA* | *ACAN* | *ACAN* | *ACAN* |
| *ADA2* | *ADA2* | *ACD* | *ACD* | *ACD* |
| *ADAM17* | *ADAM17* | *ACP5* | *ACP5* | *ACP5* |
| *ADAR* | *ADAR* | *ACTB* | *ACTB* | *ACTB* |
| *AICDA* | *AICDA* | *ACTN1* | *ACTN1* | *ACTN1* |
| *AIRE* | *AIRE* | *ADA* | *ADA* | *ADA* |
| *AK2* | *AK2* | *ADA2* | *ADA2* | *ADA2* |
| *AP3B1* | *ALG6* | *ADAM17* | *ADAM17* | *ADAM17* |
| *ATM* | *ANGPT1* | *ADAMTS13* | *ADAMTS13* | *ADAMTS13* |
| *B2M* | *ANKZF1* | *ADAR* | *ADAR* | *ADAR* |
| *BCL10* | *AP3B1* | *ADGRE2* | *ADGRE2* | *ADGRE2* |
| *BLNK* | *AP3D1* | *AICDA* | *AICDA* | *AICDA* |
| *BLOC1S6* | *ARHGEF1* | *AIRE* | *AIRE* | *AIRE* |
| *BTK* | *ARPC1B* | *AK2* | *AK2* | *AK2* |
| *CARD11* | *ASAH1* | *AK7* | *AK7* | *AK7* |
| *CARD14* | *ATM* | *ALAS2* | *ALAS2* | *ALAS2* |
| *CARD9* | *ATP6AP1* | *ALG6* | *ALG6* | *ALG6* |
| *CASP10* | *B2M* | *ALPK1* | *ALPK1* | *ALPK1* |
| *CASP8* | *BACH2* | *ANGPT1* | *ANGPT1* | *ANGPT1* |
| *CD247* | *BCL10* | *ANKRD26* | *ANKRD26* | *ANKRD26* |
| *CD27* | *BCL11B* | *ANKZF1* | *ANKZF1* | *ANKZF1* |
| *CD3D* | *BLM* | *ANO6* | *ANO6* | *ANO6* |
| *CD3E* | *BLNK* | *AP3B1* | *AP3B1* | *AP3B1* |
| *CD3G* | *BLOC1S3* | *AP3D1* | *AP3D1* | *AP3D1* |
| *CD40LG* | *BLOC1S6* | *ARHGEF1* | *ARHGEF1* | *ARHGEF1* |
| *CD79A* | *BTK* | *ARMC4* | *ARMC4* | *ARMC4* |
| *CD79B* | *C17orf62* | *ARPC1B* | *ARPC1B* | *ARPC1B* |
| *CD8A* | *C1QA* | *ASAH1* | *ASAH1* | *ASAH1* |
| *CEBPE* | *C1QB* | *ATM* | *ATM* | *ATM* |
| *CHD7* | *C1QC* | *ATP6AP1* | *ATP6AP1* | *ATP6AP1* |
| *CIITA* | *C1S* | *ATR* | *ATR* | *ATR* |
| *CLPB* | *C2* | *B2M* | *B2M* | *B2M* |
| *COPA* | *C3* | *BACH2* | *BACH2* | *BACH2* |
| *CORO1A* | *C5* | *BCL10* | *BCL10* | *BCL10* |
| *CR2* | *C6* | *BCL11B* | *BCL11B* | *BCL11B* |
| *CSF2RA* | *C7* | *BLM* | *BLM* | *BLM* |
| *CSF3R* | *C8A* | *BLNK* | *BLNK* | *BLNK* |
| *CTC1* | *C8B* | *BLOC1S3* | *BLOC1S3* | *BLOC1S3* |
| *CTLA4* | *C9* | *BLOC1S6* | *BLOC1S6* | *BLOC1S6* |
| *CTPS1* | *CARD11* | *BRCA1* | *BRCA1* | *BRCA1* |
| *CTSC* | *CARD14* | *BRCA2* | *BRCA2* | *BRCA2* |
| *CXCR4* | *CARD8* | *BRIP1* | *BRIP1* | *BRIP1* |
| *CYBA* | *CARD9* | *BTK* | *BTK* | *BTK* |
| *CYBB* | *CARMIL2* | *C11orf70* | *C1QA* | *C1QA* |
| *DCLRE1B* | *CASP10* | *C15orf41* | *C1QB* | *C1QB* |
| *DCLRE1C* | *CASP8* | *C17orf62* | *C1QC* | *C1QC* |
| *DKC1* | *CBL* | *C1QA* | *C1S* | *C1S* |
| *DNMT3B* | *CCBE1* | *C1QB* | *C2* | *C2* |
| *DOCK2* | *CD19* | *C1QC* | *C3* | *C3* |
| *DOCK8* | *CD247* | *C1S* | *C5* | *C5* |
| *ELANE* | *CD27* | *C2* | *C6* | *C6* |
| *EPG5* | *CD3D* | *C3* | *C7* | *C7* |
| *FADD* | *CD3E* | *C5* | *C8A* | *C8A* |
| *FAS* | *CD3G* | *C6* | *C8B* | *C8B* |
| *FASLG* | *CD40* | *C7* | *C9* | *C9* |
| *FERMT3* | *CD40LG* | *C8A* | *CARD11* | *CARD11* |
| *FOXN1* | *CD46* | *C8B* | *CARD14* | *CARD14* |
| *FOXP3* | *CD55* | *C9* | *CARD8* | *CARD8* |
| *FPR1* | *CD59* | *CARD11* | *CARD9* | *CARD9* |
| *G6PC3* | *CD79A* | *CARD14* | *CARMIL2* | *CARMIL2* |
| *GATA2* | *CD79B* | *CARD8* | *CASP10* | *CASP10* |
| *GFI1* | *CD81* | *CARD9* | *CASP8* | *CASP8* |
| *HAX1* | *CD8A* | *CARMIL2* | *CBL* | *CBL* |
| *ICOS* | *CDC42* | *CASP10* | *CCBE1* | *CCBE1* |
| *IFIH1* | *CDCA7* | *CASP8* | *CCDC103* | *CCDC103* |
| *IFNGR1* | *CEBPE* | *CBL* | *CCDC114* | *CCDC114* |
| *IFNGR2* | *CFB* | *CCBE1* | *CCDC151* | *CCDC151* |
| *IGLL1* | *CFD* | *CCDC103* | *CCDC39* | *CCDC39* |
| *IKBKB* | *CFH* | *CCDC114* | *CCDC40* | *CCDC40* |
| *IL10* | *CFI* | *CCDC151* | *CCDC65* | *CCDC65* |
| *IL10RA* | *CFP* | *CCDC39* | *CCNO* | *CCNO* |
| *IL10RB* | *CHD7* | *CCDC40* | *CD19* | *CD19* |
| *IL12B* | *CIB1* | *CCDC65* | *CD247* | *CD247* |
| *IL12RB1* | *CIITA* | *CCNO* | *CD27* | *CD27* |
| *IL17F* | *CLCN7* | *CD19* | *CD3D* | *CD3D* |
| *IL17RA* | *CLPB* | *CD247* | *CD3E* | *CD3E* |
| *IL17RC* | *COL7A1* | *CD27* | *CD3G* | *CD3G* |
| *IL1RN* | *COPA* | *CD3D* | *CD40* | *CD40* |
| *IL21* | *CORO1A* | *CD3E* | *CD40LG* | *CD40LG* |
| *IL21R* | *CR2* | *CD3G* | *CD46* | *CD46* |
| *IL2RA* | *CSF2RA* | *CD40* | *CD55* | *CD55* |
| *IL2RG* | *CSF2RB* | *CD40LG* | *CD59* | *CD59* |
| *IL36RN* | *CSF3R* | *CD46* | *CD79A* | *CD79A* |
| *IL7R* | *CTC1* | *CD55* | *CD79B* | *CD79B* |
| *IRAK4* | *CTLA4* | *CD59* | *CD81* | *CD81* |
| *IRF7* | *CTPS1* | *CD79A* | *CD8A* | *CD8A* |
| *IRF8* | *CTSC* | *CD79B* | *CDAN1* | *CDAN1* |
| *ISG15* | *CXCR2* | *CD81* | *CDC42* | *CDC42* |
| *ITCH* | *CXCR4* | *CD8A* | *CDCA7* | *CDCA7* |
| *ITGB2* | *CYBA* | *CDAN1* | *CDIN1* | *CDIN1* |
| *ITK* | *CYBB* | *CDC42* | *CEBPE* | *CEBPE* |
| *JAGN1* | *CYP27A1* | *CDCA7* | *CEP164* | *CEP164* |
| *JAK3* | *DCLRE1C* | *CEBPE* | *CFAP298* | *CFAP298* |
| *LAMTOR2* | *DDX58* | *CEP164* | *CFAP300* | *CFAP300* |
| *LCK* | *DEF6* | *CFAP298* | *CFB* | *CFB* |
| *LIG4* | *DGAT1* | *CFB* | *CFD* | *CFD* |
| *LPIN2* | *DIAPH1* | *CFD* | *CFH* | *CFH* |
| *LRBA* | *DKC1* | *CFH* | *CFI* | *CFI* |
| *LYST* | *DNAJC21* | *CFI* | *CFP* | *CFP* |
| *MAGT1* | *DNASE1L3* | *CFP* | *CFTR* | *CFTR* |
| *MALT1* | *DNASE2* | *CFTR* | *CHD7* | *CHD7* |
| *MAP3K14* | *DNMT3B* | *CHD7* | *CHEK2* | *CHEK2* |
| *MEFV* | *DOCK2* | *CHEK2* | *CIB1* | *CIB1* |
| *MOGS* | *DOCK8* | *CIB1* | *CIITA* | *CIITA* |
| *MVK* | *DSG1* | *CIITA* | *CLCN7* | *CLCN7* |
| *MYD88* | *DTNBP1* | *CLCN7* | *CLPB* | *CLPB* |
| *NBN* | *DUOX2* | *CLPB* | *COL7A1* | *COL7A1* |
| *NCF2* | *EFL1* | *COL7A1* | *COPA* | *COPA* |
| *NCF4* | *EIF2AK3* | *COPA* | *CORO1A* | *CORO1A* |
| *NFAT5* | *ELANE* | *CORO1A* | *CR2* | *CR2* |
| *NFKB2* | *EPG5* | *CR2* | *CSF2RA* | *CSF2RA* |
| *NFKBIA* | *ERBIN* | *CSF2RA* | *CSF2RB* | *CSF2RB* |
| *NHEJ1* | *ERCC2* | *CSF2RB* | *CSF3R* | *CSF3R* |
| *NHP2* | *ERCC3* | *CSF3R* | *CTC1* | *CTC1* |
| *NLRC4* | *ERCC6L2* | *CTC1* | *CTLA4* | *CTLA4* |
| *NLRP12* | *EXTL3* | *CTLA4* | *CTPS1* | *CTPS1* |
| *NLRP3* | *FADD* | *CTPS1* | *CTSC* | *CTSC* |
| *NOD2* | *FANCA* | *CTSC* | *CXCR2* | *CXCR2* |
| *NOP10* | *FANCB* | *CXCR2* | *CXCR4* | *CXCR4* |
| *ORAI1* | *FANCE* | *CXCR4* | *CYBA* | *CYBA* |
| *PARN* | *FANCF* | *CYBA* | *CYBB* | *CYBB* |
| *PGM3* | *FANCI* | *CYBB* | *CYBC1* | *CYBC1* |
| *PIK3CD* | *FANCL* | *CYCS* | *CYCS* | *CYCS* |
| *PIK3R1* | *FAS* | *CYP27A1* | *CYP27A1* | *CYP27A1* |
| *PLCG2* | *FASLG* | *DBR1* | *DBR1* | *DBR1* |
| *PMM2* | *FAT4* | *DCLRE1C* | *DCLRE1C* | *DCLRE1C* |
| *PNP* | *FCHO1* | *DDX41* | *DDX41* | *DDX41* |
| *POLE* | *FERMT1* | *DDX58* | *DDX58* | *DDX58* |
| *PRF1* | *FERMT3* | *DEF6* | *DEF6* | *DEF6* |
| *PRKCD* | *FOXI3* | *DGAT1* | *DGAT1* | *DGAT1* |
| *PRKDC* | *FOXN1* | *DGKE* | *DGKE* | *DGKE* |
| *PSMB8* | *FOXP3* | *DIAPH1* | *DIAPH1* | *DIAPH1* |
| *PSTPIP1* | *FPR1* | *DKC1* | *DKC1* | *DKC1* |
| *PTPRC* | *G6PC* | *DNAAF1* | *DNAAF1* | *DNAAF1* |
| *RAB27A* | *G6PC3* | *DNAAF2* | *DNAAF2* | *DNAAF2* |
| *RAC2* | *G6PD* | *DNAAF3* | *DNAAF3* | *DNAAF3* |
| *RAG1* | *GATA2* | *DNAAF4* | *DNAAF4* | *DNAAF4* |
| *RAG2* | *GFI1* | *DNAAF5* | *DNAAF5* | *DNAAF5* |
| *RBCK1* | *GINS1* | *DNAH1* | *DNAH1* | *DNAH1* |
| *RFX5* | *GTF2E2* | *DNAH11* | *DNAH11* | *DNAH11* |
| *RFXANK* | *GTF2H5* | *DNAH5* | *DNAH5* | *DNAH5* |
| *RFXAP* | *GUCY2C* | *DNAH8* | *DNAH8* | *DNAH8* |
| *RHOH* | *HAX1* | *DNAH9* | *DNAH9* | *DNAH9* |
| *RMRP* | *HELLS* | *DNAI1* | *DNAI1* | *DNAI1* |
| *RNASEH2A* | *HMOX1* | *DNAI2* | *DNAI2* | *DNAI2* |
| *RNASEH2B* | *HPS1* | *DNAJB13* | *DNAJB13* | *DNAJB13* |
| *RNASEH2C* | *HPS3* | *DNAJC21* | *DNAJC21* | *DNAJC21* |
| *RORC* | *HPS4* | *DNAL1* | *DNAL1* | *DNAL1* |
| *RTEL1* | *HPS5* | *DNASE1L3* | *DNASE1L3* | *DNASE1L3* |
| *SAMHD1* | *HPS6* | *DNASE2* | *DNASE2* | *DNASE2* |
| *SEMA3E* | *HTRA2* | *DNMT3B* | *DNMT3B* | *DNMT3B* |
| *SH2D1A* | *HYOU1* | *DOCK2* | *DOCK2* | *DOCK2* |
| *SH3BP2* | *ICOS* | *DOCK8* | *DOCK8* | *DOCK8* |
| *SLC29A3* | *ICOSLG* | *DRC1* | *DRC1* | *DRC1* |
| *SLC35C1* | *IFIH1* | *DSG1* | *DSG1* | *DSG1* |
| *SLC37A4* | *IFNAR1* | *DTNBP1* | *DTNBP1* | *DTNBP1* |
| *SLC7A7* | *IFNAR2* | *DUOX2* | *DUOX2* | *DUOX2* |
| *SMARCAL1* | *IFNGR1* | *EFL1* | *EFL1* | *EFL1* |
| *SP110* | *IFNGR2* | *EIF2AK3* | *EIF2AK3* | *EIF2AK3* |
| *SPINK5* | *IGLL1* | *ELANE* | *ELANE* | *ELANE* |
| *STAT1* | *IKBKB* | *EPCAM* | *EPCAM* | *EPCAM* |
| *STAT2* | *IL10* | *EPG5* | *EPG5* | *EPG5* |
| *STAT3* | *IL10RA* | *ERBIN* | *ERBIN* | *ERBIN* |
| *STAT5B* | *IL10RB* | *ERCC2* | *ERCC2* | *ERCC2* |
| *STIM1* | *IL12B* | *ERCC3* | *ERCC3* | *ERCC3* |
| *STK4* | *IL12RB1* | *ERCC4* | *ERCC4* | *ERCC4* |
| *STX11* | *IL12RB2* | *ERCC6L2* | *ERCC6L2* | *ERCC6L2* |
| *STXBP2* | *IL17F* | *ETV6* | *ETV6* | *ETV6* |
| *TAP1* | *IL17RA* | *EXTL3* | *EXTL3* | *EXTL3* |
| *TAP2* | *IL17RC* | *FADD* | *FADD* | *FADD* |
| *TAPBP* | *IL1RN* | *FANCA* | *FANCA* | *FANCA* |
| *TAZ* | *IL21* | *FANCB* | *FANCB* | *FANCB* |
| *TBX1* | *IL21R* | *FANCC* | *FANCC* | *FANCC* |
| *TCN2* | *IL23R* | *FANCD2* | *FANCD2* | *FANCD2* |
| *TERC* | *IL2RA* | *FANCE* | *FANCE* | *FANCE* |
| *TERT* | *IL2RB* | *FANCF* | *FANCF* | *FANCF* |
| *TICAM1* | *IL2RG* | *FANCG* | *FANCG* | *FANCG* |
| *TINF2* | *IL36RN* | *FANCI* | *FANCI* | *FANCI* |
| *TLR3* | *IL6R* | *FANCL* | *FANCL* | *FANCL* |
| *TMC6* | *IL6ST* | *FANCM* | *FANCM* | *FANCM* |
| *TMC8* | *IL7R* | *FAS* | *FAS* | *FAS* |
| *TMEM173* | *IRAK4* | *FASLG* | *FASLG* | *FASLG* |
| *TNFRSF13B* | *IRF2BP2* | *FAT4* | *FAT4* | *FAT4* |
| *TNFRSF13C* | *IRF4* | *FCHO1* | *FCHO1* | *FCHO1* |
| *TNFRSF1A* | *IRF7* | *FERMT1* | *FERMT1* | *FERMT1* |
| *TNFRSF4* | *IRF8* | *FERMT3* | *FERMT3* | *FERMT3* |
| *TNFSF12* | *IRF9* | *FLI1* | *FLI1* | *FLI1* |
| *TPP2* | *ISG15* | *FNIP1* | *FNIP1* | *FNIP1* |
| *TRAF3* | *ITCH* | *FOXI3* | *FOXI3* | *FOXI3* |
| *TRAF3IP2* | *ITGAM* | *FOXN1* | *FOXN1* | *FOXN1* |
| *TREX1* | *ITGB2* | *FOXP3* | *FOXP3* | *FOXP3* |
| *TRNT1* | *ITK* | *FPR1* | *FPR1* | *FPR1* |
| *TTC7A* | *JAGN1* | *G6PC* | *G6PC* | *G6PC* |
| *TYK2* | *JAK1* | *G6PC3* | *G6PC3* | *G6PC3* |
| *UNC13D* | *JAK3* | *G6PD* | *G6PD* | *G6PD* |
| *UNC93B1* | *KDM6A* | *GAS8* | *GAS8* | *GAS8* |
| *UNG* | *KMT2A* | *GATA1* | *GATA1* | *GATA1* |
| *VPS13B* | *KMT2D* | *GATA2* | *GATA2* | *GATA2* |
| *VPS45* | *LAMTOR2* | *GFI1* | *GFI1* | *GFI1* |
| *WAS* | *LAT* | *GINS1* | *GINS1* | *GINS1* |
| *WIPF1* | *LCK* | *GLRX5* | *GLRX5* | *GLRX5* |
| *XIAP* | *LCT* | *GP1BA* | *GP1BA* | *GP1BA* |
| *ZAP70* | *LIG1* | *GP6* | *GP6* | *GP6* |
| *ZBTB24* | *LIG4* | *GP9* | *GP9* | *GP9* |
|  | *LIPA* | *GTF2E2* | *GTF2E2* | *GTF2E2* |
|  | *LPIN2* | *GTF2H5* | *GTF2H5* | *GTF2H5* |
|  | *LRBA* | *GUCY2C* | *GUCY2C* | *GUCY2C* |
|  | *LRRC8A* | *HAX1* | *HAX1* | *HAX1* |
|  | *LYN* | *HELLS* | *HELLS* | *HELLS* |
|  | *LYST* | *HMOX1* | *HMOX1* | *HMOX1* |
|  | *MAGT1* | *HPS1* | *HPS1* | *HPS1* |
|  | *MALT1* | *HPS3* | *HPS3* | *HPS3* |
|  | *MAP3K14* | *HPS4* | *HPS4* | *HPS4* |
|  | *MCM4* | *HPS5* | *HPS5* | *HPS5* |
|  | *MEFV* | *HPS6* | *HPS6* | *HPS6* |
|  | *MKL1* | *HTRA2* | *HTRA2* | *HTRA2* |
|  | *MOGS* | *HYOU1* | *HYOU1* | *HYOU1* |
|  | *MPLKIP* | *ICOS* | *ICOS* | *ICOS* |
|  | *MS4A1* | *ICOSLG* | *ICOSLG* | *ICOSLG* |
|  | *MSN* | *IFIH1* | *IFIH1* | *IFIH1* |
|  | *MTHFD1* | *IFNAR1* | *IFNAR1* | *IFNAR1* |
|  | *MVK* | *IFNAR2* | *IFNAR2* | *IFNAR2* |
|  | *MYD88* | *IFNGR1* | *IFNGR1* | *IFNGR1* |
|  | *MYO5B* | *IFNGR2* | *IFNGR2* | *IFNGR2* |
|  | *MYSM1* | *IGLL1* | *IGLL1* | *IGLL1* |
|  | *NBAS* | *IKBKB* | *IKBKB* | *IKBKB* |
|  | *NBN* | *IKZF1* | *IKZF1* | *IKZF1* |
|  | *NCF2* | *IL10* | *IL10* | *IL10* |
|  | *NCF4* | *IL10RA* | *IL10RA* | *IL10RA* |
|  | *NCSTN* | *IL10RB* | *IL10RB* | *IL10RB* |
|  | *NEUROG3* | *IL12B* | *IL12B* | *IL12B* |
|  | *NFAT5* | *IL12RB1* | *IL12RB1* | *IL12RB1* |
|  | *NFE2L2* | *IL12RB2* | *IL12RB2* | *IL12RB2* |
|  | *NFKB1* | *IL17F* | *IL17F* | *IL17F* |
|  | *NFKB2* | *IL17RA* | *IL17RA* | *IL17RA* |
|  | *NFKBIA* | *IL17RC* | *IL17RC* | *IL17RC* |
|  | *NHEJ1* | *IL18BP* | *IL18BP* | *IL18BP* |
|  | *NHP2* | *IL1RN* | *IL1RN* | *IL1RN* |
|  | *NLRC4* | *IL21* | *IL21* | *IL21* |
|  | *NLRP1* | *IL21R* | *IL21R* | *IL21R* |
|  | *NLRP12* | *IL23R* | *IL23R* | *IL23R* |
|  | *NLRP3* | *IL2RA* | *IL2RA* | *IL2RA* |
|  | *NOD2* | *IL2RB* | *IL2RB* | *IL2RB* |
|  | *NOP10* | *IL2RG* | *IL2RG* | *IL2RG* |
|  | *NSMCE3* | *IL36RN* | *IL36RN* | *IL36RN* |
|  | *OAS1* | *IL6R* | *IL6R* | *IL6R* |
|  | *ORAI1* | *IL6ST* | *IL6ST* | *IL6ST* |
|  | *OSTM1* | *IL7R* | *IL7R* | *IL7R* |
|  | *OTULIN* | *IRAK4* | *IRAK4* | *IRAK4* |
|  | *PARN* | *IRF2BP2* | *IRF2BP2* | *IRF2BP2* |
|  | *PAX1* | *IRF4* | *IRF4* | *IRF4* |
|  | *PEPD* | *IRF7* | *IRF7* | *IRF7* |
|  | *PGM3* | *IRF8* | *IRF8* | *IRF8* |
|  | *PIK3CD* | *IRF9* | *IRF9* | *IRF9* |
|  | *PIK3R1* | *ISG15* | *ISG15* | *ISG15* |
|  | *PLCG2* | *ITCH* | *ITCH* | *ITCH* |
|  | *PMM2* | *ITGA2B* | *ITGA2B* | *ITGA2B* |
|  | *PNP* | *ITGAM* | *ITGAM* | *ITGAM* |
|  | *POLA1* | *ITGB2* | *ITGB2* | *ITGB2* |
|  | *POLD1* | *ITGB3* | *ITGB3* | *ITGB3* |
|  | *POLE* | *ITK* | *ITK* | *ITK* |
|  | *POLE2* | *JAGN1* | *JAGN1* | *JAGN1* |
|  | *POLR3A* | *JAK1* | *JAK1* | *JAK1* |
|  | *POMP* | *JAK2* | *JAK2* | *JAK2* |
|  | *PRF1* | *JAK3* | *JAK3* | *JAK3* |
|  | *PRKCD* | *KAT6A* | *KAT6A* | *KAT6A* |
|  | *PRKDC* | *KDM1A* | *KDM1A* | *KDM1A* |
|  | *PSENEN* | *KDM6A* | *KDM6A* | *KDM6A* |
|  | *PSMA3* | *KIF23* | *KIF23* | *KIF23* |
|  | *PSMB4* | *KIT* | *KIT* | *KIT* |
|  | *PSMB8* | *KLF1* | *KLF1* | *KLF1* |
|  | *PSMG2* | *KLHDC8B* | *KLHDC8B* | *KLHDC8B* |
|  | *PSTPIP1* | *KMT2A* | *KMT2A* | *KMT2A* |
|  | *PTPRC* | *KMT2D* | *KMT2D* | *KMT2D* |
|  | *RAB27A* | *LAMTOR2* | *LAMTOR2* | *LAMTOR2* |
|  | *RAC2* | *LARS2* | *LARS2* | *LARS2* |
|  | *RAG1* | *LAT* | *LAT* | *LAT* |
|  | *RAG2* | *LCK* | *LCK* | *LCK* |
|  | *RANBP2* | *LCT* | *LCT* | *LCT* |
|  | *RASGRP1* | *LIG1* | *LIG1* | *LIG1* |
|  | *RBCK1* | *LIG4* | *LIG4* | *LIG4* |
|  | *RELA* | *LIPA* | *LIPA* | *LIPA* |
|  | *RELB* | *LPIN2* | *LPIN2* | *LPIN2* |
|  | *RFX5* | *LRBA* | *LRBA* | *LRBA* |
|  | *RFXANK* | *LRRC56* | *LRRC56* | *LRRC56* |
|  | *RFXAP* | *LRRC6* | *LRRC6* | *LRRC6* |
|  | *RHOH* | *LRRC8A* | *LRRC8A* | *LRRC8A* |
|  | *RIPK1* | *LYN* | *LYN* | *LYN* |
|  | *RMRP* | *LYST* | *LYST* | *LYST* |
|  | *RNASEH2A* | *MAD2L2* | *MAD2L2* | *MAD2L2* |
|  | *RNASEH2B* | *MAGT1* | *MAGT1* | *MAGT1* |
|  | *RNASEH2C* | *MALT1* | *MALT1* | *MALT1* |
|  | *RNF113A* | *MAP3K14* | *MAP3K14* | *MAP3K14* |
|  | *RNF168* | *MBD4* | *MBD4* | *MBD4* |
|  | *RNF31* | *MCIDAS* | *MCIDAS* | *MCIDAS* |
|  | *RNU4ATAC* | *MCM4* | *MCM4* | *MCM4* |
|  | *RORC* | *MECOM* | *MECOM* | *MECOM* |
|  | *RPSA* | *MEFV* | *MEFV* | *MEFV* |
|  | *RTEL1* | *MKL1* | *MKL1* | *MKL1* |
|  | *SAMD9* | *MLH1* | *MLH1* | *MLH1* |
|  | *SAMD9L* | *MOGS* | *MOGS* | *MOGS* |
|  | *SAMHD1* | *MPL* | *MPL* | *MPL* |
|  | *SAR1B* | *MPLKIP* | *MPLKIP* | *MPLKIP* |
|  | *SCO2* | *MS4A1* | *MS4A1* | *MS4A1* |
|  | *SEC61A1* | *MSH2* | *MSH2* | *MSH2* |
|  | *SEMA3E* | *MSH6* | *MSH6* | *MSH6* |
|  | *SERPING1* | *MSN* | *MSN* | *MSN* |
|  | *SH2D1A* | *MTHFD1* | *MTHFD1* | *MTHFD1* |
|  | *SH3BP2* | *MVK* | *MVK* | *MVK* |
|  | *SH3KBP1* | *MYD88* | *MYD88* | *MYD88* |
|  | *SI* | *MYH9* | *MYH9* | *MYH9* |
|  | *SIAE* | *MYO5B* | *MYO5B* | *MYO5B* |
|  | *SKIV2L* | *MYSM1* | *MYSM1* | *MYSM1* |
|  | *SLC26A3* | *NAF1* | *NAF1* | *NAF1* |
|  | *SLC29A3* | *NBAS* | *NBAS* | *NBAS* |
|  | *SLC35C1* | *NBN* | *NBN* | *NBN* |
|  | *SLC37A4* | *NCF2* | *NCF2* | *NCF1* |
|  | *SLC39A7* | *NCF4* | *NCF4* | *NCF2* |
|  | *SLC46A1* | *NCKAP1L* | *NCKAP1L* | *NCF4* |
|  | *SLC5A1* | *NCSTN* | *NCSTN* | *NCKAP1L* |
|  | *SLC7A7* | *NDUFB11* | *NDUFB11* | *NCSTN* |
|  | *SLC9A3* | *NEUROG3* | *NEUROG3* | *NDUFB11* |
|  | *SLX4* | *NF1* | *NF1* | *NEUROG3* |
|  | *SMARCAL1* | *NFAT5* | *NFAT5* | *NF1* |
|  | *SMARCD2* | *NFE2L2* | *NFE2L2* | *NFAT5* |
|  | *SNX10* | *NFKB1* | *NFKB1* | *NFE2L2* |
|  | *SP110* | *NFKB2* | *NFKB2* | *NFKB1* |
|  | *SPINK5* | *NFKBIA* | *NFKBIA* | *NFKB2* |
|  | *SPINT2* | *NHEJ1* | *NHEJ1* | *NFKBIA* |
|  | *SPPL2A* | *NHP2* | *NHP2* | *NHEJ1* |
|  | *SRP54* | *NLRC4* | *NLRC4* | *NHP2* |
|  | *SRP72* | *NLRP1* | *NLRP1* | *NLRC4* |
|  | *STAT1* | *NLRP12* | *NLRP12* | *NLRP1* |
|  | *STAT2* | *NLRP3* | *NLRP3* | *NLRP12* |
|  | *STAT3* | *NME8* | *NME8* | *NLRP3* |
|  | *STAT4* | *NOD2* | *NOD2* | *NME8* |
|  | *STAT5B* | *NOP10* | *NOP10* | *NOD2* |
|  | *STIM1* | *NOTCH2* | *NOTCH2* | *NOP10* |
|  | *STK4* | *NPAT* | *NPAT* | *NOTCH2* |
|  | *STN1* | *NSMCE3* | *NSMCE3* | *NPAT* |
|  | *STX11* | *OAS1* | *OAS1* | *NSMCE3* |
|  | *STX3* | *OFD1* | *OFD1* | *OAS1* |
|  | *STXBP2* | *ORAI1* | *ORAI1* | *OFD1* |
|  | *TAOK2* | *OSTM1* | *OSTM1* | *ORAI1* |
|  | *TAP1* | *OTULIN* | *OTULIN* | *OSTM1* |
|  | *TAP2* | *P2RY12* | *P2RY12* | *OTULIN* |
|  | *TAPBP* | *PALB2* | *PALB2* | *P2RY12* |
|  | *TAZ* | *PARN* | *PARN* | *PALB2* |
|  | *TBX1* | *PAX1* | *PAX1* | *PARN* |
|  | *TCF3* | *PEPD* | *PEPD* | *PAX1* |
|  | *TCIRG1* | *PGM3* | *PGM3* | *PEPD* |
|  | *TCN2* | *PIH1D3* | *PIH1D3* | *PGM3* |
|  | *TERC* | *PIK3CD* | *PIK3CD* | *PIH1D3* |
|  | *TERT* | *PIK3R1* | *PIK3R1* | *PIK3CD* |
|  | *TFRC* | *PLA2G4A* | *PLA2G4A* | *PIK3R1* |
|  | *TGFB1* | *PLCG2* | *PLCG2* | *PLA2G4A* |
|  | *TGFBR1* | *PLG* | *PLG* | *PLCG2* |
|  | *TGFBR2* | *PLVAP* | *PLVAP* | *PLG* |
|  | *THBD* | *PMM2* | *PMM2* | *PLVAP* |
|  | *TICAM1* | *PMS2* | *PMS2* | *PMM2* |
|  | *TIMM50* | *PNLIP* | *PNLIP* | *PMS2* |
|  | *TINF2* | *PNP* | *PNP* | *PNLIP* |
|  | *TLR3* | *POLA1* | *POLA1* | *PNP* |
|  | *TMC6* | *POLD1* | *POLD1* | *POLA1* |
|  | *TMC8* | *POLD2* | *POLD2* | *POLD1* |
|  | *TMEM173* | *POLE* | *POLE* | *POLD2* |
|  | *TNFAIP3* | *POLE2* | *POLE2* | *POLE* |
|  | *TNFRSF11A* | *POLR3A* | *POLR3A* | *POLE2* |
|  | *TNFRSF13B* | *POLR3F* | *POLR3F* | *POLR3A* |
|  | *TNFRSF13C* | *POMP* | *POMP* | *POLR3F* |
|  | *TNFRSF1A* | *POT1* | *POT1* | *POMP* |
|  | *TNFRSF4* | *PRF1* | *PRF1* | *POT1* |
|  | *TNFRSF6B* | *PRKCD* | *PRKCD* | *PRF1* |
|  | *TNFRSF9* | *PRKDC* | *PRKDC* | *PRKCD* |
|  | *TNFSF11* | *PSENEN* | *PSENEN* | *PRKDC* |
|  | *TNFSF12* | *PSMA3* | *PSMA3* | *PSENEN* |
|  | *TONSL* | *PSMB4* | *PSMB4* | *PSMA3* |
|  | *TOP2B* | *PSMB8* | *PSMB8* | *PSMB4* |
|  | *TP63* | *PSMG2* | *PSMG2* | *PSMB8* |
|  | *TPP2* | *PSTPIP1* | *PSTPIP1* | *PSMG2* |
|  | *TRAF3* | *PTEN* | *PTEN* | *PSTPIP1* |
|  | *TRAF3IP2* | *PTPRC* | *PTPRC* | *PTEN* |
|  | *TREX1* | *PUS1* | *PUS1* | *PTPRC* |
|  | *TRNT1* | *RAB27A* | *RAB27A* | *PUS1* |
|  | *TTC37* | *RAC2* | *RAC2* | *RAB27A* |
|  | *TTC7A* | *RAD51* | *RAD51* | *RAC2* |
|  | *TYK2* | *RAD51C* | *RAD51C* | *RAD51* |
|  | *UNC13D* | *RAG1* | *RAG1* | *RAD51C* |
|  | *UNC45A* | *RAG2* | *RAG2* | *RAG1* |
|  | *UNC93B1* | *RANBP2* | *RANBP2* | *RAG2* |
|  | *UNG* | *RASGRP1* | *RASGRP1* | *RANBP2* |
|  | *USB1* | *RASGRP2* | *RASGRP2* | *RASGRP1* |
|  | *VAV1* | *RBCK1* | *RBCK1* | *RASGRP2* |
|  | *VPS13B* | *RBM8A* | *RBM8A* | *RBCK1* |
|  | *VPS45* | *RECQL4* | *RECQL4* | *RBM8A* |
|  | *WAS* | *REL* | *REL* | *RECQL4* |
|  | *WDR1* | *RELA* | *RELA* | *REL* |
|  | *WIPF1* | *RELB* | *RELB* | *RELA* |
|  | *WRAP53* | *RFWD3* | *RFWD3* | *RELB* |
|  | *XIAP* | *RFX5* | *RFX5* | *RFWD3* |
|  | *ZAP70* | *RFXANK* | *RFXANK* | *RFX5* |
|  | *ZBTB24* | *RFXAP* | *RFXAP* | *RFXANK* |
|  | *ZCCHC8* | *RHOH* | *RHOH* | *RFXAP* |
|  | *ZNF341* | *RIPK1* | *RIPK1* | *RHOH* |
|  |  | *RMRP* | *RMRP* | *RIPK1* |
|  |  | *RNASEH2A* | *RNASEH2A* | *RMRP* |
|  |  | *RNASEH2B* | *RNASEH2B* | *RNASEH2A* |
|  |  | *RNASEH2C* | *RNASEH2C* | *RNASEH2B* |
|  |  | *RNF113A* | *RNF113A* | *RNASEH2C* |
|  |  | *RNF168* | *RNF168* | *RNF113A* |
|  |  | *RNF31* | *RNF31* | *RNF168* |
|  |  | *RNU4ATAC* | *RNU4ATAC* | *RNF31* |
|  |  | *RORC* | *RORC* | *RNU4ATAC* |
|  |  | *RPGR* | *RPGR* | *RORC* |
|  |  | *RPL11* | *RPL11* | *RPGR* |
|  |  | *RPL15* | *RPL15* | *RPL11* |
|  |  | *RPL18* | *RPL18* | *RPL15* |
|  |  | *RPL19* | *RPL19* | *RPL18* |
|  |  | *RPL23* | *RPL23* | *RPL19* |
|  |  | *RPL26* | *RPL26* | *RPL23* |
|  |  | *RPL27* | *RPL27* | *RPL26* |
|  |  | *RPL31* | *RPL31* | *RPL27* |
|  |  | *RPL35* | *RPL35* | *RPL31* |
|  |  | *RPL35A* | *RPL35A* | *RPL35* |
|  |  | *RPL5* | *RPL5* | *RPL35A* |
|  |  | *RPL9* | *RPL9* | *RPL5* |
|  |  | *RPS10* | *RPS10* | *RPL9* |
|  |  | *RPS15A* | *RPS15A* | *RPS10* |
|  |  | *RPS19* | *RPS19* | *RPS15A* |
|  |  | *RPS24* | *RPS24* | *RPS19* |
|  |  | *RPS26* | *RPS26* | *RPS24* |
|  |  | *RPS27* | *RPS27* | *RPS26* |
|  |  | *RPS28* | *RPS28* | *RPS27* |
|  |  | *RPS29* | *RPS29* | *RPS28* |
|  |  | *RPS7* | *RPS7* | *RPS29* |
|  |  | *RPSA* | *RPSA* | *RPS7* |
|  |  | *RSPH1* | *RSPH1* | *RPSA* |
|  |  | *RSPH3* | *RSPH3* | *RSPH1* |
|  |  | *RSPH4A* | *RSPH4A* | *RSPH3* |
|  |  | *RSPH9* | *RSPH9* | *RSPH4A* |
|  |  | *RTEL1* | *RTEL1* | *RSPH9* |
|  |  | *RUNX1* | *RUNX1* | *RTEL1* |
|  |  | *SAMD9* | *SAMD9* | *RUNX1* |
|  |  | *SAMD9L* | *SAMD9L* | *SAMD9* |
|  |  | *SAMHD1* | *SAMHD1* | *SAMD9L* |
|  |  | *SAR1B* | *SAR1B* | *SAMHD1* |
|  |  | *SBF2* | *SBF2* | *SAR1B* |
|  |  | *SCO2* | *SCO2* | *SBF2* |
|  |  | *SEC23B* | *SEC23B* | *SCO2* |
|  |  | *SEC61A1* | *SEC61A1* | *SEC23B* |
|  |  | *SEMA3E* | *SEMA3E* | *SEC61A1* |
|  |  | *SERPING1* | *SERPING1* | *SEMA3E* |
|  |  | *SGPL1* | *SGPL1* | *SERPING1* |
|  |  | *SH2D1A* | *SH2D1A* | *SGPL1* |
|  |  | *SH3BP2* | *SH3BP2* | *SH2D1A* |
|  |  | *SH3KBP1* | *SH3KBP1* | *SH3BP2* |
|  |  | *SI* | *SI* | *SH3KBP1* |
|  |  | *SIAE* | *SIAE* | *SI* |
|  |  | *SKIV2L* | *SKIV2L* | *SIAE* |
|  |  | *SLC10A2* | *SLC10A2* | *SKIV2L* |
|  |  | *SLC19A2* | *SLC19A2* | *SLC10A2* |
|  |  | *SLC25A38* | *SLC25A38* | *SLC19A2* |
|  |  | *SLC26A3* | *SLC26A3* | *SLC25A38* |
|  |  | *SLC29A3* | *SLC29A3* | *SLC26A3* |
|  |  | *SLC35C1* | *SLC35C1* | *SLC29A3* |
|  |  | *SLC37A4* | *SLC37A4* | *SLC35C1* |
|  |  | *SLC39A7* | *SLC39A7* | *SLC37A4* |
|  |  | *SLC46A1* | *SLC46A1* | *SLC39A7* |
|  |  | *SLC51B* | *SLC51B* | *SLC46A1* |
|  |  | *SLC5A1* | *SLC5A1* | *SLC51B* |
|  |  | *SLC7A7* | *SLC7A7* | *SLC5A1* |
|  |  | *SLC9A3* | *SLC9A3* | *SLC7A7* |
|  |  | *SLX4* | *SLX4* | *SLC9A3* |
|  |  | *SMARCAL1* | *SMARCAL1* | *SLX4* |
|  |  | *SMARCD2* | *SMARCD2* | *SMARCAL1* |
|  |  | *SNX10* | *SNX10* | *SMARCD2* |
|  |  | *SP110* | *SP110* | *SNX10* |
|  |  | *SPAG1* | *SPAG1* | *SP110* |
|  |  | *SPINK5* | *SPINK5* | *SPAG1* |
|  |  | *SPINT2* | *SPINT2* | *SPINK5* |
|  |  | *SPPL2A* | *SPPL2A* | *SPINT2* |
|  |  | *SRP54* | *SRP54* | *SPPL2A* |
|  |  | *SRP72* | *SRP72* | *SRP54* |
|  |  | *STAT1* | *STAT1* | *SRP72* |
|  |  | *STAT2* | *STAT2* | *STAT1* |
|  |  | *STAT3* | *STAT3* | *STAT2* |
|  |  | *STAT4* | *STAT4* | *STAT3* |
|  |  | *STAT5B* | *STAT5B* | *STAT4* |
|  |  | *STIM1* | *STAT6* | *STAT5B* |
|  |  | *STK4* | *STIM1* | *STAT6* |
|  |  | *STN1* | *STK4* | *STIM1* |
|  |  | *STX11* | *STN1* | *STK4* |
|  |  | *STX3* | *STX11* | *STN1* |
|  |  | *STXBP2* | *STX3* | *STX11* |
|  |  | *TAOK2* | *STXBP2* | *STX3* |
|  |  | *TAP1* | *TAOK2* | *STXBP2* |
|  |  | *TAP2* | *TAP1* | *TAOK2* |
|  |  | *TAPBP* | *TAP2* | *TAP1* |
|  |  | *TAZ* | *TAPBP* | *TAP2* |
|  |  | *TBX1* | *TAZ* | *TAPBP* |
|  |  | *TBXA2R* | *TBX1* | *TAZ* |
|  |  | *TCF3* | *TBXA2R* | *TBX1* |
|  |  | *TCIRG1* | *TCF3* | *TBXA2R* |
|  |  | *TCN2* | *TCIRG1* | *TCF3* |
|  |  | *TERC* | *TCN2* | *TCIRG1* |
|  |  | *TERF2IP* | *TERC* | *TCN2* |
|  |  | *TERT* | *TERF2IP* | *TERC* |
|  |  | *TET2* | *TERT* | *TERF2IP* |
|  |  | *TFRC* | *TET2* | *TERT* |
|  |  | *TGFB1* | *TFRC* | *TET2* |
|  |  | *TGFBR1* | *TGFB1* | *TFRC* |
|  |  | *TGFBR2* | *TGFBR1* | *TGFB1* |
|  |  | *THBD* | *TGFBR2* | *TGFBR1* |
|  |  | *THPO* | *THBD* | *TGFBR2* |
|  |  | *TICAM1* | *THPO* | *THBD* |
|  |  | *TIMM50* | *TICAM1* | *THPO* |
|  |  | *TINF2* | *TIMM50* | *TICAM1* |
|  |  | *TLR3* | *TINF2* | *TIMM50* |
|  |  | *TLR7* | *TLR3* | *TINF2* |
|  |  | *TMC6* | *TLR7* | *TLR3* |
|  |  | *TMC8* | *TMC6* | *TLR7* |
|  |  | *TMEM173* | *TMC8* | *TMC6* |
|  |  | *TMPRSS15* | *TMEM173* | *TMC8* |
|  |  | *TNFAIP3* | *TMPRSS15* | *TMEM173* |
|  |  | *TNFRSF11A* | *TNFAIP3* | *TMPRSS15* |
|  |  | *TNFRSF13B* | *TNFRSF11A* | *TNFAIP3* |
|  |  | *TNFRSF13C* | *TNFRSF13B* | *TNFRSF11A* |
|  |  | *TNFRSF1A* | *TNFRSF13C* | *TNFRSF13B* |
|  |  | *TNFRSF4* | *TNFRSF1A* | *TNFRSF13C* |
|  |  | *TNFRSF6B* | *TNFRSF4* | *TNFRSF1A* |
|  |  | *TNFRSF9* | *TNFRSF6B* | *TNFRSF4* |
|  |  | *TNFSF11* | *TNFRSF9* | *TNFRSF6B* |
|  |  | *TNFSF12* | *TNFSF11* | *TNFRSF9* |
|  |  | *TONSL* | *TNFSF12* | *TNFSF11* |
|  |  | *TOP2B* | *TONSL* | *TNFSF12* |
|  |  | *TP53* | *TOP2B* | *TONSL* |
|  |  | *TP63* | *TP53* | *TOP2B* |
|  |  | *TPP2* | *TP63* | *TP53* |
|  |  | *TRAF3* | *TPP2* | *TP63* |
|  |  | *TRAF3IP2* | *TRAF3* | *TPP2* |
|  |  | *TREX1* | *TRAF3IP2* | *TRAF3* |
|  |  | *TRNT1* | *TREX1* | *TRAF3IP2* |
|  |  | *TSR2* | *TRNT1* | *TREX1* |
|  |  | *TTC37* | *TSR2* | *TRNT1* |
|  |  | *TTC7A* | *TTC37* | *TSR2* |
|  |  | *TUBB1* | *TTC7A* | *TTC37* |
|  |  | *TYK2* | *TUBB1* | *TTC7A* |
|  |  | *UBE2T* | *TYK2* | *TUBB1* |
|  |  | *UNC13D* | *UBE2T* | *TYK2* |
|  |  | *UNC45A* | *UNC13D* | *UBE2T* |
|  |  | *UNC93B1* | *UNC45A* | *UNC13D* |
|  |  | *UNG* | *UNC93B1* | *UNC45A* |
|  |  | *USB1* | *UNG* | *UNC93B1* |
|  |  | *VAV1* | *USB1* | *UNG* |
|  |  | *VIPAS39* | *VAV1* | *USB1* |
|  |  | *VPS13B* | *VIPAS39* | *VAV1* |
|  |  | *VPS33B* | *VPS13B* | *VIPAS39* |
|  |  | *VPS45* | *VPS33B* | *VPS13B* |
|  |  | *WAS* | *VPS45* | *VPS33B* |
|  |  | *WDR1* | *WAS* | *VPS45* |
|  |  | *WIPF1* | *WDR1* | *WAS* |
|  |  | *WNT2B* | *WIPF1* | *WDR1* |
|  |  | *WRAP53* | *WNT2B* | *WIPF1* |
|  |  | *XIAP* | *WRAP53* | *WNT2B* |
|  |  | *XRCC2* | *XIAP* | *WRAP53* |
|  |  | *YARS2* | *XRCC2* | *XIAP* |
|  |  | *ZAP70* | *YARS2* | *XRCC2* |
|  |  | *ZBTB24* | *ZAP70* | *YARS2* |
|  |  | *ZCCHC8* | *ZBTB24* | *ZAP70* |
|  |  | *ZMYND10* | *ZCCHC8* | *ZBTB24* |
|  |  | *ZNF341* | *ZMYND10* | *ZCCHC8* |
|  |  |  | *ZNF341* | *ZMYND10* |
|  |  |  |  | *ZNF341* |
